# The biogeography of the mucosa-associated microbiome in health and disease

**DOI:** 10.3389/fmicb.2024.1454910

**Published:** 2024-10-14

**Authors:** Peter R. Sternes, Ayesha Shah, Camila Ayala Pintos, Thomas Fairlie, Natasha Koloski, Seungha Kang, Kaylyn D. Tousignant, Simon J. McIlroy, Mark Morrison, Gene W. Tyson, Gerald J. Holtmann

**Affiliations:** ^1^Centre for Microbiome Research, School of Biomedical Sciences, Queensland University of Technology (QUT), Translational Research Institute, Woolloongabba, QLD, Australia; ^2^Department of Gastroenterology and Hepatology, Princess Alexandra Hospital, Brisbane, QLD, Australia; ^3^Faculty of Medicine, University of Queensland, Brisbane, QLD, Australia; ^4^Faculty of Health and Behavioural Sciences, University of Queensland, Brisbane, QLD, Australia; ^5^Frazer Institute, Faculty of Medicine, University of Queensland, Woolloongabba, QLD, Australia

**Keywords:** microbiome, proton pump inhibitor, symptom severity, Crohn’s disease, ulcerative colitis, gut-brain

## Abstract

**Introduction:**

Little is known about the biogeography of the mucosa associated microbiome (MAM) in patients with inflammatory bowel disease (IBD) versus controls in different segments of the gastrointestinal tract, as well as the links between the MAM, gastrointestinal symptoms, and use of proton pump inhibitors (PPI).

**Methods:**

We recruited 59 controls (without structural abnormalities and gastrointestinal symptoms), 44 patients with ulcerative colitis (UC) and 31 with Crohn’s disease (CD). Biopsies from various segments of the upper and lower gastrointestinal tract were collected. Microbial composition was assessed via 16S rRNA gene amplicon analysis and the bacterial load of the mucosal biopsies were assessed via qPCR. The MAM was examined in the context of disease status, PPI usage, the severity of gastrointestinal symptoms, and the symptom response to a standardised nutrient challenge (SNC).

**Results:**

Microbial communities of the MAM in the upper and lower gastrointestinal tract differed. IBD patients were characterised by relative and absolute depletion of numerous genera known to produce butyrate and/or propionate, with the largest differentiation being the depletion of Faecalibacterium in the lower gastrointestinal tract of CD patients. Notably, PPI users exhibited an enrichment of Faecalibacterium in the lower gastrointestinal tract. The severity of gastrointestinal symptoms, as well as the symptom response to the SNC, were significantly associated with MAM composition in the gastrointestinal tract.

**Conclusion:**

The absolute and relative composition of the MAM is variable across different segments of the gastrointestinal tract. These quantitative changes indicates that MAM can be targeted in specific segments of the GI tract to improve patient outcomes.

## Introduction

Microorganisms colonising the human gastrointestinal (GI) tract play a crucial role in host physiological functions, such as the digestion of food, absorption of micronutrients, production of vitamins (Resta, 2009), maintenance of immune homeostasis (Wu and Wu, 2012) and intestinal barrier function (Camilleri et al., 2012). Thus, “microbial dysbiosis” of the gut, defined as the alterations in the composition, density, and function of the intestinal microbiome, is associated with the pathogenesis of both intestinal and extra-intestinal disorders.

Given the harsh environment, with a low pH and/or high concentrations of digestive enzymes and bile acids, the upper GI tract harbours a relatively small number of bacteria compared to that of the lower GI tract (Shah et al., 2018), however the implications for health and disease might be substantial due to the large surface area (Helander and Fändriks, 2014).

In addition to this longitudinal microbiota composition variation along the GI tract, there is also latitudinal microbial variation, where different intestinal compartments harbour unique microbial communities (Donaldson et al., 2016). The intestinal epithelium is separated from the lumen by a thick and physiochemically complex mucus layer, and there are significant differences between the microbiota in the intestinal lumen, attached and embedded within the mucus layer, and in immediate proximity of the epithelium (Shanahan et al., 2016). Facilitated by human metagenome sequencing efforts, the exploration of dysregulated relationships between the microbiota and its host has advanced our comprehension on how compositional variations of these populations play a role in diseases such as disorders of gut-brain interaction (DGBI) and inflammatory bowel disease (IBD). Recently, our team have shown that duodenal mucosal bacterial load was significantly greater in patients with DGBI as compared to those with IBD and controls (Shah et al., 2020). Thus, the absolute quantity of microorganisms is also an important element to consider when studying the mucosa associated microbiome (MAM) (Vandeputte et al., 2017).

Extraintestinal disorders such as anxiety, depression, and chronic pain reportedly affect ~30% of IBD patients (Neuendorf et al., 2016). Although it remains speculative, observational studies suggest a relationship between psychological morbidity, presence of self-reported IBD symptoms and/or inflammatory activity. Psychological disorders may influence the function of the GI tract via eliciting stress responses, increasing the secretion of intestinal stress hormones which may stimulate inflammatory cytokines and elicit pro-inflammatory effects, and increasing intestinal permeability. Conversely, the intestinal permeability may allow the gut microbiota or microbial products to interact with the nervous system, thus this relationship might be bidirectional (Koloski et al., 2012, 2016). Animal models have highlighted links between visceral hypersensitivity and psychological disorders (Clouse et al., 2006; Johnson et al., 2012). The aetiology of visceral hypersensitivity in DGBI and IBD is uncertain, but this mechanism may also be relevant for this disease. The possibility that the “gut-brain axis” bi-directionally influences psychological health and the progression of IBD in a manner that highlights the importance of considering this potential mechanism when studying IBD.

In this context, there is a need to complement our current understanding of the gut microbiome, which is mainly based on faecal community profiling, with profiling of the mucosa-associated community along the GI tract to better understand its contribution to human health and disease. The relatively easy access and high microbial biomass level of faecal samples makes them an “attractive” resource for study. However, the faecal microbiome cannot account for the spatial distribution of microbes along the GI tract and is largely influenced by diet and intestinal transit. In addition, increasing evidence indicates that the gastrointestinal microbiome is influenced by medications that suppress gastric acid secretion, such as proton pump inhibitors (PPI), however further research is required as evidence of their ability to modify the MAM in the various segments of the GI tract are thus far limited and controversial (Bruno et al., 2019).

It follows, that the present study aims to: (a) compare the biogeography of the MAM (in terms of relative and absolute abundance) at different gut segments of individuals with IBD (Crohn’s disease (CD) and Ulcerative colitis (UC)) and controls; (b) identify the influence of PPI on microbial colonisation along the gastrointestinal tract; and (c) identify the links between the MAM, gastrointestinal symptoms and extraintestinal symptoms.

## Materials and methods

### Subject recruitment and study design

A summary of the sampled cohort is provided in Tables 1, 2, with expanded versions available in Supplementary Tables S1, S2. This study was approved by the institutional ethics committee (Research Protocol HREC/13/QPAH/690). After obtaining informed written consent, we recruited 75 patients with IBD (44 UC, 31 CD) and 59 control subjects between May 2016 and June 2019 at a single tertiary hospital outpatient clinic in Brisbane, Australia. From this group, 50 individuals (18 CD, 32 controls) only underwent upper endoscopy, 65 underwent only lower endoscopy (10 CD, 39 UC, 16 controls), and 19 (3 CD, 5 UC, 11 controls) provided samples from both the lower and upper GI by undergoing same day upper endoscopy and colonoscopy (Table 2). The control group included patients with no relevant gastrointestinal symptoms but referred for investigation of a positive faecal occult blood test (FOBT) and/or iron deficiency (ID) with or without anaemia. The inclusion of these persons in the study required no relevant gastrointestinal symptoms and normal endoscopic findings. Exclusion criteria included active IBD, any other organic or functional gastrointestinal conditions, psychiatric disease, and antibiotic and/or probiotic usage within the 2 months prior to sampling. All study subjects were negative for *Helicobacter pylori* as confirmed by histology. The diagnosis of IBD was based upon a combination of endoscopic, histopathological, biochemical and radiological investigations, according to established clinical standards (Okobi et al., 2021), with all IBD patients being in clinical remission, as assessed utilising a combination of clinical assessment and tests including serum albumin, C-reactive protein, and faecal calprotectin, endoscopic findings, and the Mayo Risk Score (Schroeder et al., 1987) or the Crohn’s disease activity index (Best et al., 1976). All recruited subjects were interviewed by a study nurse to assess their dietary history, and all participants adhered to a non-specialised Western-style diet.

**Table 1:** Cohort demographics.

Groups	Control (*n* = 59)	UC (*n* = 44)	CD (*n* = 31)	FDR-adj. *p*
Demographic characteristics				
Age (years)*	56.90 (±1.77)	45 (±2.16)	42.71(±2.24)	C vs. UC = 0.00001* C vs. CD = 0.00009* UC vs. CD = 0.464
Gender (Female), *n* (%)	28 (47.46%)	22 (50%)	20 (64.52%)	NS
BMI (kg/m^2^) *	27.84 (±0.80)	28.82 (±0.81)	27.75(±1.28)	C vs. UC = 0.399 C vs. CD = 0.953 UC vs. CD = 0.494
Gastrointestinal symptoms				
Total SAGIS score*	4.84 (±0.64)	11.91 (±1.29)	18.90(±2.92)	C vs. UC = 0.00002* C vs. CD = 0.00008* UC vs. CD = 0.034*
Nutrient challenge score	109.125	181.8947	147.5	C vs. UC = 0.9788 C vs. CD = 0.5508 UC vs. CD = 0.7147
Bacterial load, 16S rRNA: beta-actin				
Rectum bacterial load*	0.028(±0.008)	0.025(±0.006)	0.107(±0.096)	C vs. UC = 0.749 C vs. CD = 0.494 UC vs. CD = 0.48
Right colon bacterial load*	0.058(±0.020)	0.027(±0.011)	0.003(±0.0005)	C vs. UC = 0.219 C vs. CD = 0.048* UC vs. CD = 0.05
Terminal ileum bacterial load*	0.138 (±0.031)	0.152 (±0.027)	0.071 (±0.043)	C vs. UC = 0.372 C vs. CD = 0.731 UC vs. CD = 0.44
Duodenum bacterial load*	0.074 (±0.014)	0.019 (±0.003)	0.031 (±0.008)	C vs. UC = 0.001* C vs. CD = 0.022 * UC vs. CD = 0.174
Medication				
PPI, *n* (%)	9 (15.25%)	7 (15.91%)	3 (9.68%)	NS

^*^Values expressed as Mean (± SEM). Student *t*-test and fisher’s exact test where applicable. UC, Ulcerative colitis; CD, Crohn’s Disease; BMI, Basal metabolic index; SAGIS, Structured assessment of gastrointestinal symptoms.

**Table 2:** Summary of the number of samples collected from various segments of the gastrointestinal tract.

Sites	Total	CD	UC	CONTROL	PPI	NO PPI	SAGIS	NCT
TI	77	12	41	24	8	68	77	37
RC	46	3	22	21	6	40	46	24
R	54	4	28	22	6	48	54	28
DU	67	21	5	41	11	56	67	31
O	51	21	0	30	11	40	51	21
G	52	20	0	32	11	41	52	23
Only UPPER (DU, O, G)	50	18	0	32	11	39	50	20
Only LOWER (TI, RC, R)	65	10	39	16	8	56	65	31
LOWER & UPPER	19	3	5	11	0	19	19	10
ALL PATIENTS	134	31	44	59	19	115	134	61

UC, ulcerative colitis; CD, Crohn’s disease; TI, terminal ileum; RC, right colon; R, rectum; DU, duodenum; O, oesophagus; G, gastric, PPI, proton pump inhibitors, NCT, nutrient challenge test, SAGIS, structured assessment of gastrointestinal symptoms questionnaire.

### Collection of clinical data

Clinical data and demographic information were obtained from the state-wide integrated Electronic Medical Record (iEMR). All clinical data (including tests initiated by any health care providers) available on the system were reviewed to confirm/establish the patient’s diagnosis.

### Assessment of gastrointestinal and extraintestinal symptoms

The severity of gastrointestinal and extraintestinal symptoms and psychiatric comorbidities were assessed utilising a validated questionnaire (SAGIS, Structured Assessment of Gastrointestinal Symptoms (Koloski et al., 2017)). This instrument consists of questions regarding five symptom domains: epigastric symptoms (meal related symptoms: postprandial pain, epigastric pain, bloating, fullness, early satiety, retrosternal discomfort, abdominal cramps), irritability, acid regurgitation, nausea/vomit, constipation, as well as questions on extraintestinal symptoms (headache, chronic fatigue, back pain, sleep disturbances, and self-reported depression and anxiety). Each symptom is scored on a 5-point Likert scale from 0 (indicating no problem) to 4 (indicating a very severe problem). The scores from each domain are summed to obtain a total SAGIS score. In this study, we focused on the analysis of total SAGIS, as well as investigating the epigastric and extraintestinal subdomains separately. For subjects referred for screening endoscopies without relevant pathology, 95% had a total SAGIS<12, 95% had an epigastric SAGIS≤4 and 95% had an extraintestinal SAGIS≤4. Thus, all subjects in the study with a total SAGIS score ≥ 12, epigastric score > 4 and extraintestinal SAGIS >4 were deemed to have a “high” score in those domains.

In addition, the gastrointestinal symptom response to a standardised nutrient challenge was captured from a subset of the recruited individuals (Table 2), as described previously (Haag et al., 2004). Briefly, after an 8-h fast, subjects were asked to drink 200 mL of a standardised enteral feeding solution (Ensure^R^) every 5 min up to a cumulative volume of 800 mL. At baseline and 5 min after each 200 mL drink, symptoms were assessed using a visual analogue scale (range 0–100 mm) with 0 = no symptom and 100 = unbearably severe.

### Endoscopic procedures

Standard endoscopic equipment (Olympus EVIS EXERA III, CV-190, Olympus irrigation pump and Olympus processor, Tokyo, Japan) were used and all procedures were documented in a clinical documentation system (Provation®, MN, United States). In addition to clinically indicated biopsies, two additional intestinal biopsies were taken from the second part of the duodenum utilising the Brisbane aseptic biopsy device (Shanahan et al., 2016), along with two biopsies from both the lower third of the oesophagus, and gastric antrum under standard conditions. Colonoscopy preparation included a clear liquid diet and bowel cleansing with 3–4 L Golytely^R^ (PEG 3350 plus electrolytes) (Kutyla et al., 2020) as split preparation with the last dose taken >3.5 h prior to the procedure. During colonoscopy, two biopsies each were taken from the terminal ileum, right ascending colon, and rectum (hereafter referred to as TI, RC, and R, respectively) utilising the standard biopsy forceps. Biopsy samples for microbiome assessment were immediately placed, under aseptic conditions, into a sterile tube containing RNA later (Qiagen, Venlo, Netherlands). Samples were incubated at room temperature for 30 min, then frozen and stored at-80°C.

### DNA extraction and bacterial load measurements from biopsies

DNA extraction was performed using a recently described modification of the repeated bead beating and column-based purification protocol developed for the recovery of microbial DNA from tissue samples (Shanahan et al., 2023). Bacterial load on individual tissue samples was assessed using a quantitative PCR (qPCR) approach following our previously published methods (Gurusamy et al., 2021). The following optimised primer sets were utilised: *β*-actin (Forward: TCCGCAAAGACCTGTACGC; Reverse: CAGTGAGGACCCTGGATGTG) and bacterial domain specific 16S rRNA (1114-Forward: CGGCAACGAGCGCAACCC; 1,221-Reverse: CATTGTAGCACGTGTGTAGCC) (Zhong et al., 2017). Standards of known copy number were constructed using serial dilutions of the pUC19 plasmid containing either the human β-actin gene or *Streptococcus* sp. 16S rRNA gene and samples were analysed on a Quantstudio 6 Real-Time PCR system. The number of copies of the β-actin and bacterial 16S rRNA genes were quantified with respect to their respective standard curves, and bacterial load expressed as a fractional value, being the ratio between 16S rRNA: β-actin gene copies.

### Library preparation and sequencing

Extracted DNA from biopsy tissues were profiled by high-throughput amplicon sequencing. The V6-V8 region of the gene encoding 16S ribosomal RNA was amplified using the primers 917-forward (GAATTGRCGGGGRCC; bacterial domain specific) and 1,392-reverse (ACGGGCGGTGWGTRC; universal), with the Q5 DNA polymerase (NEB), as previously described (Shanahan et al., 2018). The choice of hypervariable region was informed by our previous studies (Yu and Morrison, 2004), reported effectiveness in recovering key mucosa-associated bacteria (Abellan-Schneyder et al., 2021), as well as evaluation using TestPrime (Klindworth et al., 2013) against the SILVA v138 rRNA reference database (Quast et al., 2012). PCR products were purified using AMPure XP beads (Beckman Coulter). Barcoded PCR libraries (Nextera XT v2 Index Kit) were further purified (AMPure XP beads), quantified (Quantus), and pooled to 4 nM. The libraries were sequenced on an Illumina MiSeq using the MiSeq Reagent Kit v3 (2 × 300 bp), by the Australian Centre for Ecogenomics, University of Queensland.

### 16S rRNA amplicon sequence processing and statistical analysis

16S rRNA gene amplicon data were quality assessed, trimmed, and filtered using Kneaddata v0.10.0.^1^ Filtered samples were then processed using the Quantitative Insights into Microbial Ecology version 2 (QIIME2) (Bolyen et al., 2019), with Cutadapt v2.1 (Martin, 2011) and DADA2 (Callahan et al., 2016) were used for trimming and removal of chimera sequences, and merging of paired-end reads. Taxonomy was assigned based on the NR99 SILVA v138 rRNA reference database (Quast et al., 2012). Twenty-one annotated sequence variants (ASVs) were identified as contaminants and removed based on a relative abundance of greater than 1% on average across the negative (no template) controls, their occurrence in two or more controls. Sufficient decontamination of the sample was confirmed using Decontam (Davis et al., 2018). Samples were rarefied to 1,000 amplicons and samples below this threshold were excluded from further analysis. Absolute abundance values were calculated via multiplication of relative abundance values, derived from QIIME2, and the bacterial load (microbial 16S: human *β*-actin) for each of the lower GI biopsies.

Multidimensional data visualisation of beta diversity was conducted using a sparse partial least squares discriminant analysis (sPLSDA) on arcsine squared-root transformed data, as implemented in R v4.2.2 (R Core Team, 2013) as part of the MixOmics package v6.23.3 (Rohart et al., 2017). Association of the microbial composition (beta diversity) with metadata of interest was conducted using a PERMANOVA test (adonis2; nperm = 10,000) as part of vegan v2.6–4 (Dixon, 2003) on arcsine squared root transformed data at the genus level, taking into account the non-independence of repeated samples where multiple sites per individual were co-analysed, as well as the sources of covariation such as BMI, age, gender, PPI usage and disease status, where appropriate. Alpha diversity metrics were calculated by the Shannon index and tested via a Wilcoxon Rank Sum test between groups. Enrichment or depletion of specific genera was tested using the MaAsLin2 v1.7.3 (Mallick et al., 2021) package, taking into consideration covariates (typically BMI, age, gender, PPI usage and/or disease status) where appropriate, on arcsine squared root transformed data. Graphs and figures were generated using ggplot2 v3.4.0 (Wickham, 2011). For core microbiome analysis, the presence of specific genera in 50, 75 and 90% of samples at each of the sampled sites was measured.

## Results

### Biogeography of the microbial communities of the GI tract

**Figure 1:**
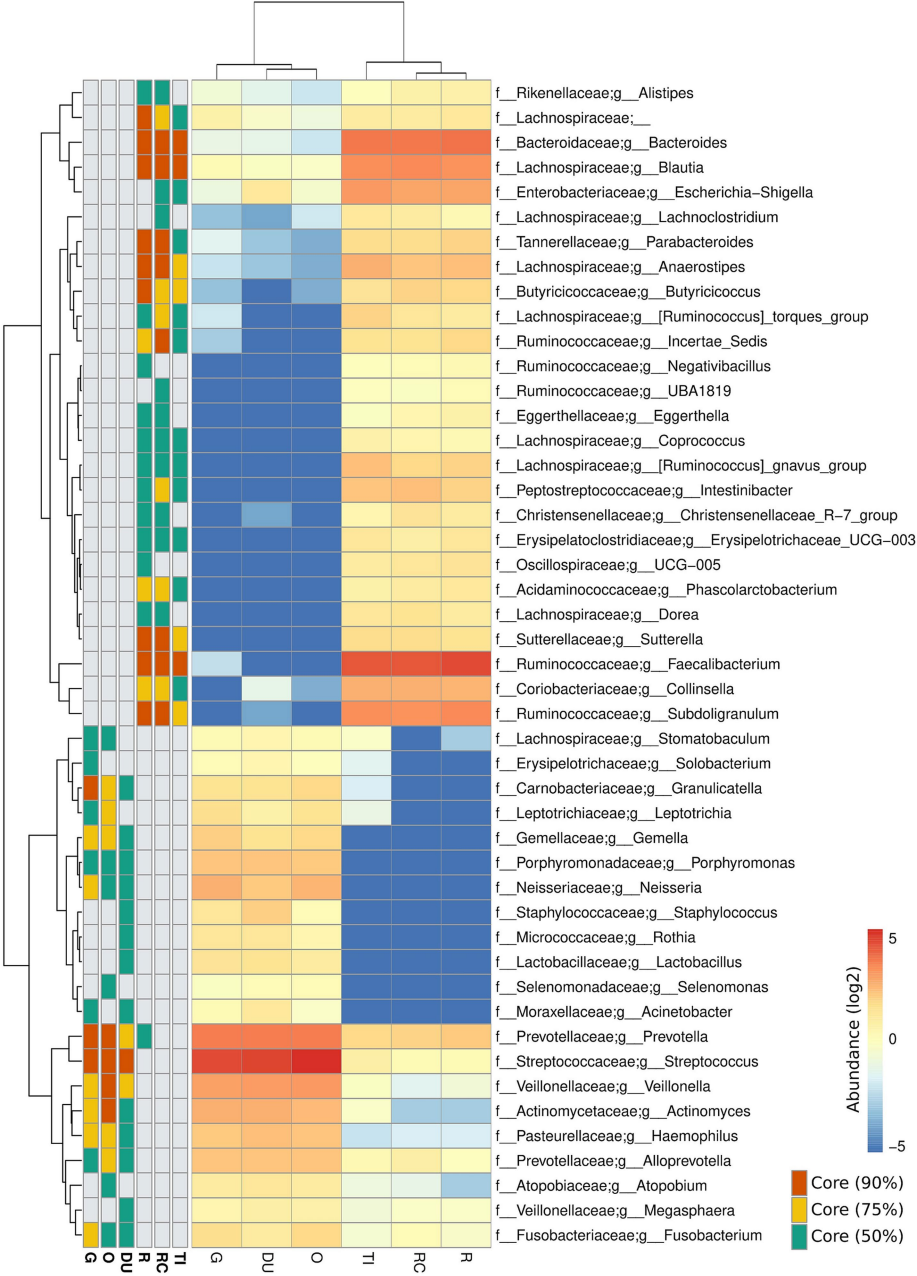
Composition of the core microbiome at each site along the lower and upper gastrointestinal tracts of controls. Heatmap values are expressed in log of the mean relative bacterial abundance from each site. Core microbiota present in 90, 75 and 50% of participants are shown. Taxa lacking a g_ prefix were unable to be assigned at the genus level and represent family-level abundance. Samples were collected from the oesophagus (O), gastric antrum (G), duodenum (DU), terminal ileum (TI), right colon (RC) and rectum (R).

Stark differences were noted in the mucosal microbial communities recovered from the tissue samples of the proximal/upper GI segments (oesophagus = O, gastric = G, and duodenum = D) compared to the distal/lower GI segments (terminal ileum = TI, right colon = RC, and rectum = R) of the gastrointestinal tract. Analysis of the core microbiome amongst controls revealed that all the proximal segments showed a high prevalence of ASVs assigned to *Streptococcus, Prevotella* and *Veillonella* (present in 90% of the samples) (Figure 1). On the other hand, the distal segments showed a high prevalence of ASVs assigned to *Faecalibacterium*, *Bacteroides and Blautia* (present in 90% of the samples). *Subdoligranulum, Anaerostipes and Sutterella* were also prevalent to a lesser degree in the lower gut (present in 75% of the samples) (Figure 1). Similar core microbiome taxa were also found in patients with IBD (Supplementary Figure S1). Due to the nature of the sampling regime, only 19 patients provided samples from both the upper and lower GI segments (Table 2). Despite the lack of overlap in patients providing samples from both segments, the broadly consistent microbiome composition across all samples is reflective of actual differences between the upper and lower GI segments, as opposed to differences in the patient cohorts.

### The MAM of UC and CD patients

**Figure 2:**
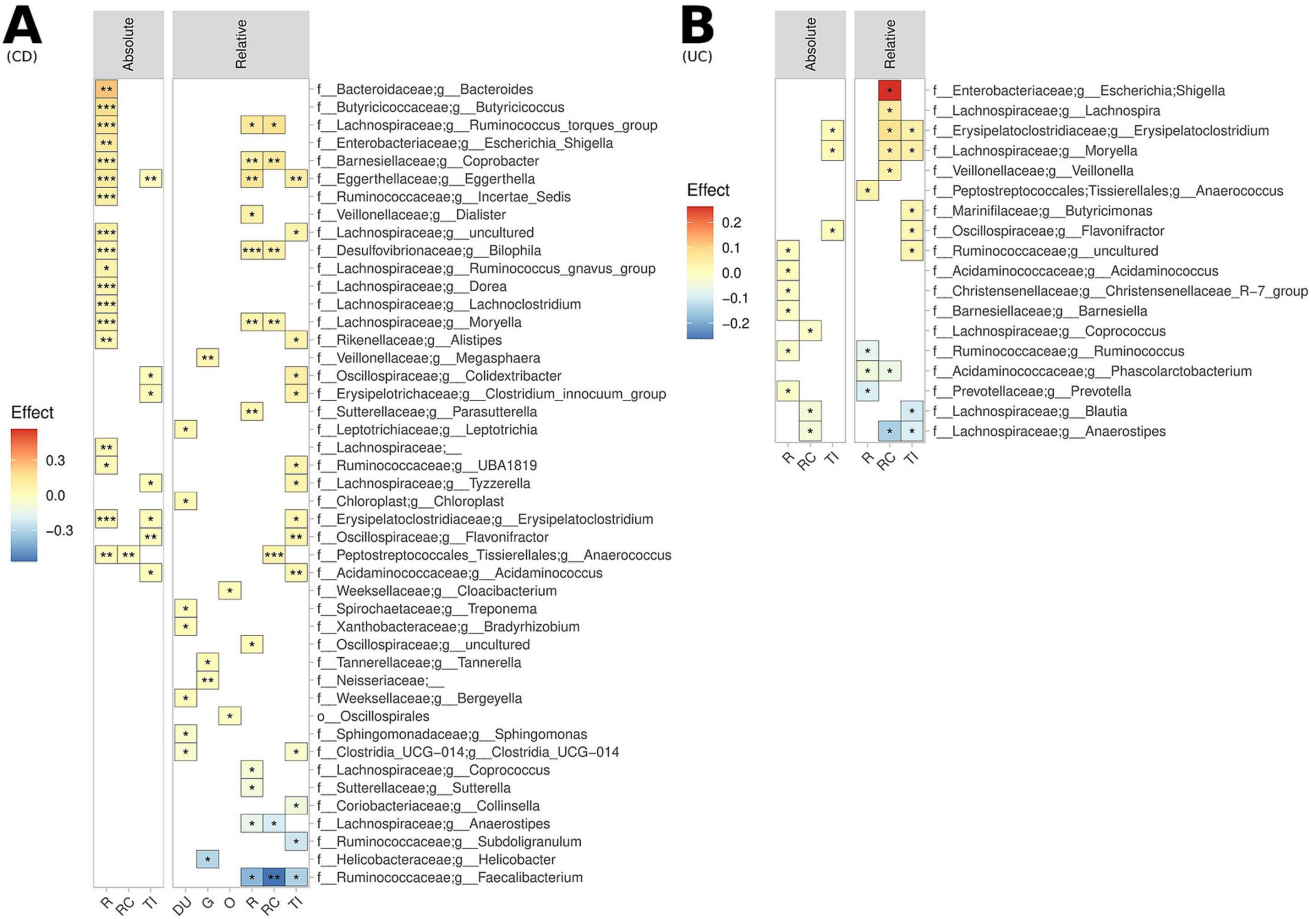
Biogeography of significantly enriched and depleted bacterial genera in **(A)** UC patients and **(B)** CD patients, relative to controls, across the lower GI tract, measured both in terms of relative abundance (upper and lower GI) and absolute abundance (lower GI). ***p < 0.001, **p < 0.01, *p < 0.05. This analysis is not FDR corrected due to sample size limitations.

**Figure 3:**
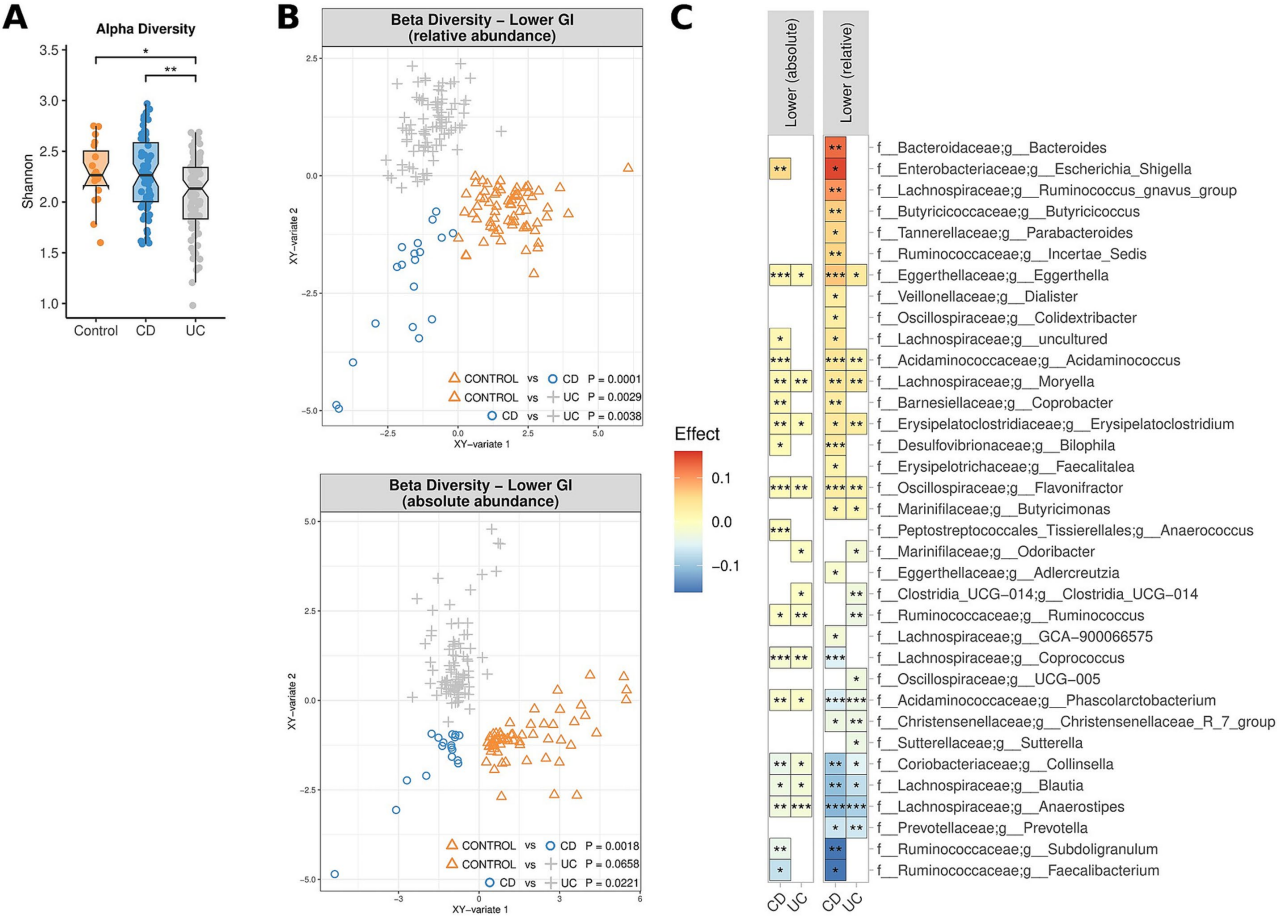
Comparison of the microbiome composition of UC patients, CD patients and controls, as measured by relative and absolute abundance (lower GI tract). **(A)** Comparison of species richness (alpha diversity) across the lower GI tract. **(B)** Multidimensional visualisation and PERMANOVA testing of microbial composition (beta diversity). Microbial composition in the upper GI tract was not significantly, thus the data is not shown. **(C)** Significantly enriched and depleted bacterial genera. All showed taxa are *p* < 0.05. ***p < 0.001, **p < 0.01, *p < 0.05.

Considering specific GI segments, substantial differences were noted for the bacterial genera enriched/depleted in CD and UC patients compared to controls, most notably a significant depletion of *Faecalibacterium* across all lower GI segments in patients with CD (Figure 2). Numerous bacterial genera also demonstrated differences in terms of absolute abundance, but not relative abundance, and *vice-versa*, demonstrating the value of assessing both metrics. To maximise sample size, all samples from lower GI segments and upper GI segments were co-analysed. Whilst there were no significant differences in the alpha diversity for controls versus UC and CD patients in the upper GI tract, there was a significant reduction in alpha diversity in the lower GI tract of patients with UC compared to controls (Figure 3A). The overall MAM composition (i.e., beta diversity) in the lower GI tract for both UC and CD patients were significantly different to those from control subjects, and with each other (control vs. CD, *p* = 0.0001; control vs. UC, *p* = 0.0029; CD vs. UC, *p* = 0.0038, Figure 3B), as well as for select taxa in terms of both relative and absolute abundances (Figure 3C). Of particular note, the MAM of UC and/or CD patients were characterised by a depletion of numerous genera which are known to produce butyrate and/or propionate. These include *Anaerostipes*, *Blautia*, *Collinsella*, *Coprococcus*, *Faecalibacterium*, *Phascolarctobacterium*, *Prevotella*, *Ruminococcus* and *Subdoligranulum.* For CD patients specifically, a strong depletion of *Faecalibacterium* (0.028 < *p* < 0.036; −0.16 < effect size<−0.07) was also noted, as well as an enrichment of *Escherichia-Shigella* (0.002 < *p* < 0.015; 0.05 < effect size<0.14). Full lists of bacterial genera and the associated *p*-values (*p* < 0.05) and effect sizes are available in Supplementary Table S3.

### The effect of PPI usage on the MAM

**Figure 4:**
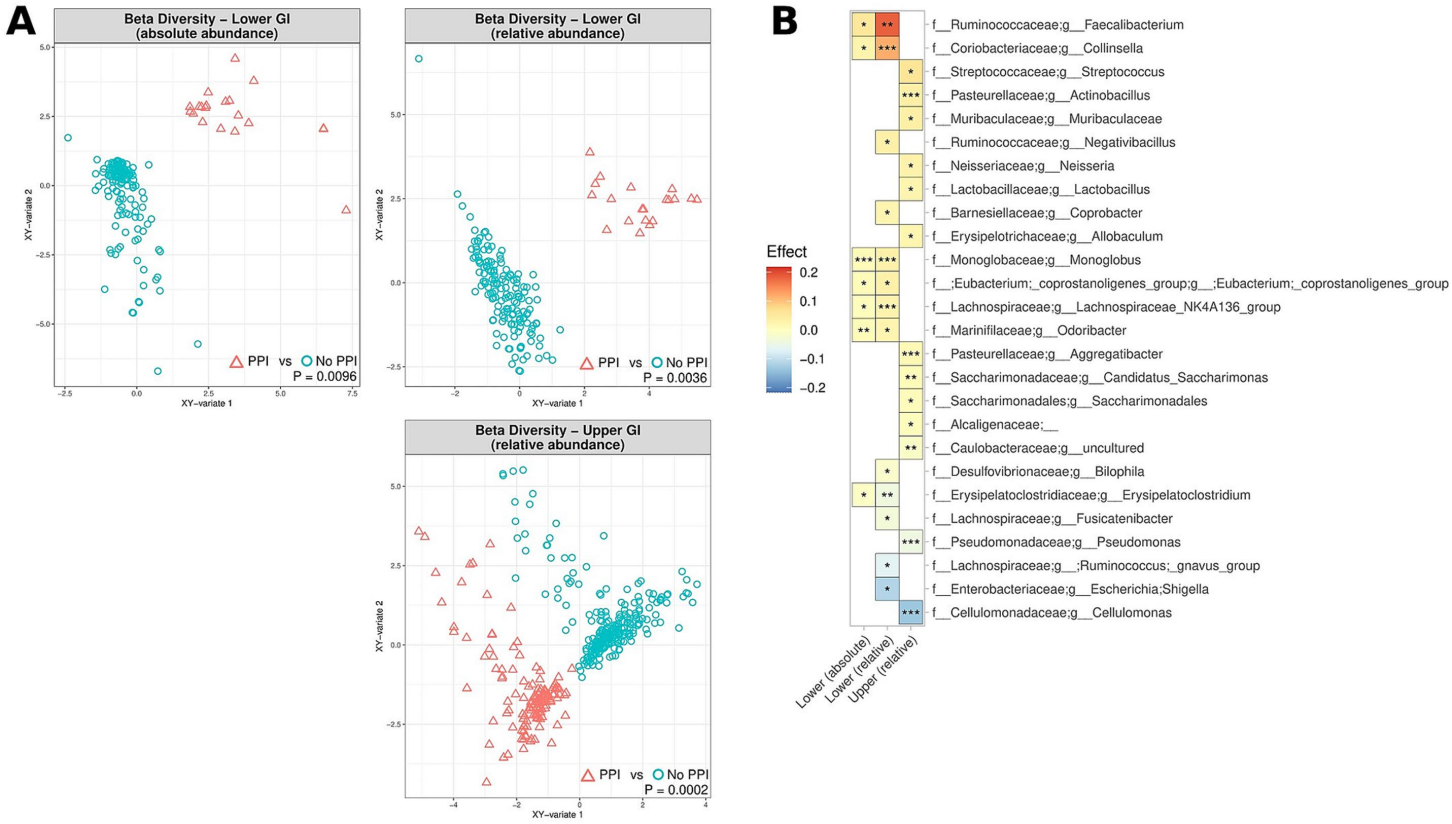
The effect of PPI usage upon microbiome composition of UC patients, CD patients and controls, measured by relative abundance (upper and lower GI) and absolute abundance (lower GI). **(A)** Multidimensional visualisation and PERMANOVA testing of microbial composition (beta diversity). **(B)** Significantly enriched and depleted bacterial genera. ***p < 0.001, **p < 0.01, *p < 0.05.

Whilst PPI therapy did not appear to affect the alpha diversity of the MAM, PPI therapy was noted to be associated with significant changes in beta diversity in both the upper and lower GI tract, both in terms of relative and absolute abundance (0.0002 < *p* < 0.0096) (Figure 4A). Of note was an absolute and relative enrichment of *Faecalibacterium* in the lower GI tract (0.0059 < *p* < 0.039) which also corresponded to the largest effect size (0.055 < effect size<0.18) amongst all the significantly differentiated genera (Figure 4B). Other changes to note include an absolute and relative enrichment of genus *Collinsella* (4.9e10^−5^ < *p* < 0.021; 0.025 < effect size <0.12) and a relative depletion of *Escherichia-Shigella* (*p* = 0.03; effect size = −0.11). Full lists of bacterial genera and the associated *p*-values (*p* < 0.05) and effect sizes are available in Supplementary Table S4.

### The MAM, symptom severity and symptom response

**Figure 5:**
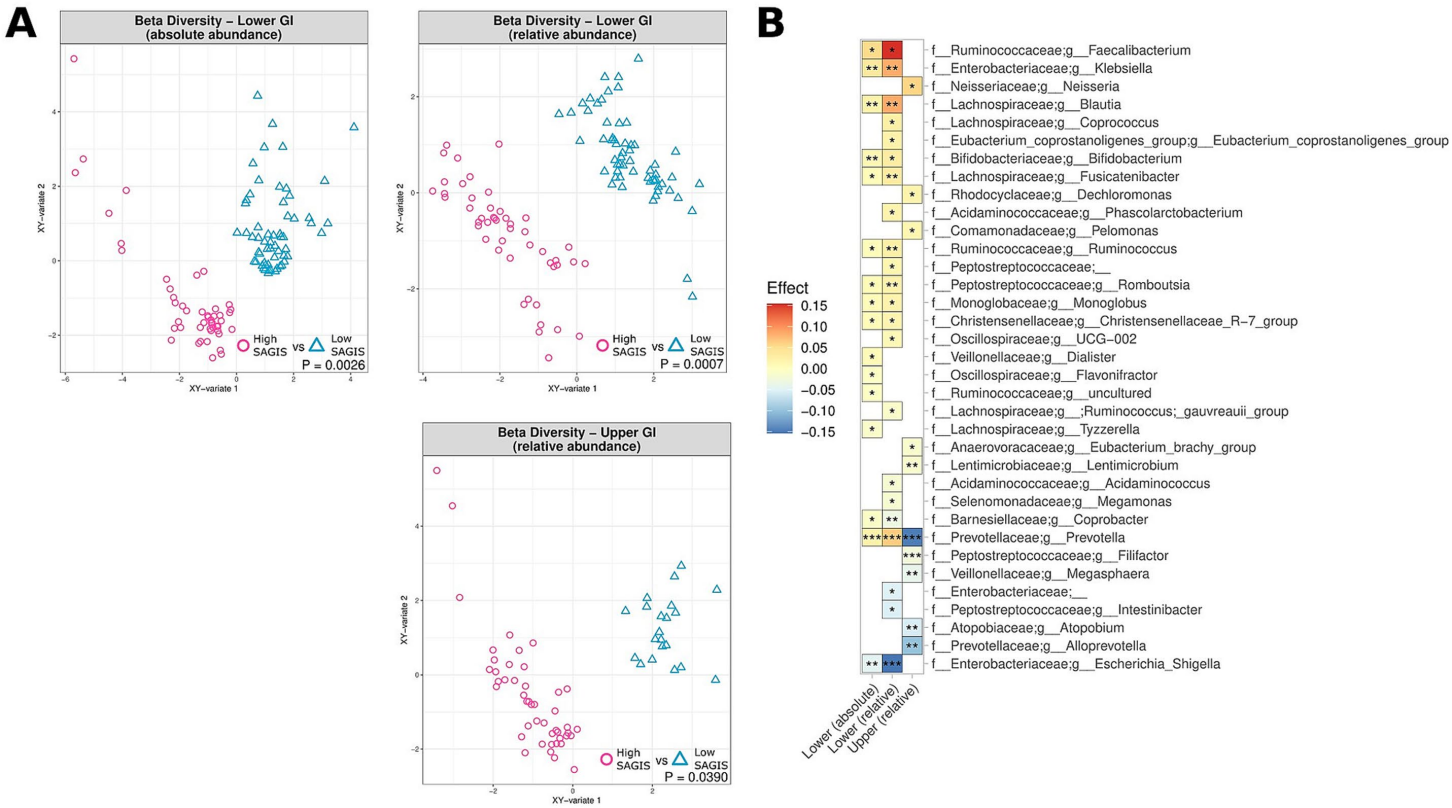
Correlation of microbiome composition in UC and CD patients and gastrointestinal symptoms, as measured by the SAGIS questionnaire, across the upper and lower GI tracts, measured in terms of relative abundance (upper and lower GI) and absolute abundance (lower GI). “High” corresponds to SAGIS scores ≥12, whereas “low” corresponds to SAGIS scores <12. **(A)** Multidimensional visualisation and PERMANOVA testing of microbial composition (beta diversity). **(B)** Significantly enriched and depleted bacterial genera in UC and CD patients with high SAGIS (≥ 12). ***p < 0.001, **p < 0.01, *p < 0.05.

Symptom severity, as assessed by the validated SAGIS questionnaire, was considered “high” in patients with a total SAGIS≥12, with 95% of controls exhibiting scores<12. Elevated symptom severity was correlated with significant differences in beta diversity of the MAM in the upper and lower GI tracts of IBD patients, both in terms of absolute and relative abundance (0.0007 < *p* < 0.039) (Figure 5A). No observable differences in alpha diversity were noted. Elevated SAGIS symptom severity was associated with an enrichment of *Faecalibacterium* (0.014 < *p* < 0.032; 0.054 < effect size<0.15), and depletion of *Escherichia-Shigella* (5e10^4^ < *p* < 0.0018; −0.15 < effect size<−0.046) in the lower GI tract, amongst many other genera (Figure 5B). Full lists of bacterial genera and the associated *p*-values (*p* < 0.05) and effect sizes are available in Supplementary Table S5.

**Figure 6:**
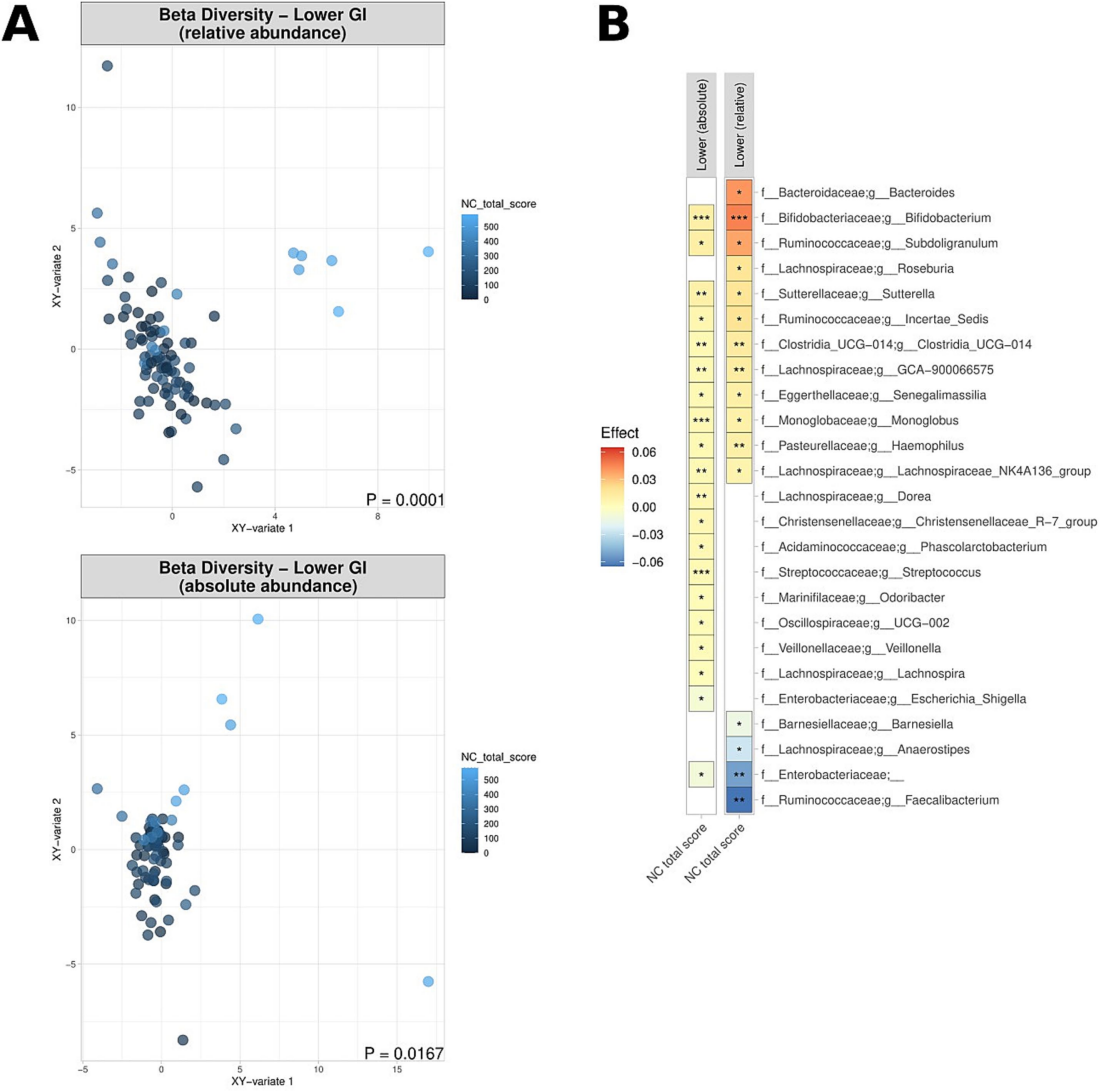
Correlation of relative and absolute microbiome compositions and nutrient challenge (NC) scores across the lower GI tract for CD patients, UC patients and non-IBD controls. **(A)** Multidimensional visualisation and PERMANOVA testing of microbial composition (beta diversity). Microbial composition in the upper GI tract was not significantly, thus the data is not shown. **(B)** Significantly enriched and depleted bacterial genera. ***p < 0.001, **p < 0.01, *p < 0.05.

The visceral symptom response to a standardised nutrient challenge test was determined. This test involves the ingestion of a nutrient solution and is a diagnostic procedure to evaluate gastrointestinal function and the response of the digestive system. Elevated scores (i.e., more severe symptoms after the nutrient challenge) were noted to correspond to significant differences in beta diversity in the lower GI tract, in terms of both absolute and relative abundance (Figure 6A), with dissimilarity metrics increasing along aside nutrient challenge scores. Similarly, increased epigastric (meal-related) sub-scores as part of the SAGIS questionnaire were also correlated with significant differences in beta diversity in the lower GI tract (Supplementary Figure S2). More severe symptoms in response to the nutrient challenge test were associated with a depletion of *Faecalibacterium* relative abundance in the lower GI tract (*p* = 0.0015; effect size = −0.05) (Figure 6B), and enrichment of *Bacteroides* and *Bifidobacterium* (1.3e^−7^ < *p* < 2.4e^−4^; 0.011 < effect size<0.046). Full lists of bacterial genera and the associated *p*-values (*p* < 0.05) and effect sizes are available in Supplementary Table S6.

## Discussion

Previous microbiome studies have primarily focused on faecal samples or studied the MAM in defined singular segments of the GI tract. The study by Gevers et al. (2014) of the treatment-naive microbiome in pediatric Crohn’s disease is an exception and suggested the taxonomic profiles from terminal ileum and rectal biopsies better reflect the microbial dysbiosis associated with disease for this cohort than stool. To the best of our knowledge, this is the first study to assess the biogeography of the MAM both in terms of the relative and absolute abundances of bacterial taxa from IBD patients and controls. Furthermore, whilst many gastrointestinal diseases are characterised by mucosal inflammation, there are so called disorders of gut-brain interaction (DGBI) with patients with macroscopically or microscopically normal mucosa reporting gastrointestinal symptoms that are frequently aggravated by meals (Duncanson et al., 2021; Shah et al., 2022). We thus also assessed the link between the MAM and gastrointestinal symptoms/symptom response to a standardised nutrient challenge test (as a measure of visceral sensory function) as well as a questionnaire for GI symptoms (SAGIS).

Our data reveal that the biogeography of MAM exhibits substantial heterogeneity between (a) patients with IBD, and controls (b) across the different regions of GI tract. In controls, while there was a clear distinction between the MAM of the upper and lower GI tract, the composition of the different segments of within the upper GI and lower GI were compositionally more similar with each other. Whilst the bowel preparation may have influenced the composition of the microbial communities, our recent data comparing the duodenal MAM in subjects undergoing same-day gastroscopy followed by colonoscopy (requiring bowel preparation) or gastroscopy alone did not reveal differences in the microbial community that could be linked to the bowel preparation (unpublished results).

Comparison of patients with IBD and controls revealed large statistically significant differences in the overall microbial composition (beta diversity) and specific taxa abundances for the lower GI tracts (Figure 2). Compared to controls, UC and CD patients were characterised by significant relative and absolute depletion of genera known to produce butyrate in the lower GI tract. In CD patients, the strongest signals found relate to a relative depletion of *Faecalibacterium* across the TI, RC, and R. *Faecalibacterium* as a genus is a compositionally abundant in health, and in addition to butyrate-production has been shown to produce other anti-inflammatory factors and promote Treg production and immune tolerance (Quévrain et al., 2016; Sokol et al., 2008). The lower gut MAM of the UC patients studied here were characterised by a relative and absolute depletion of *Anaerostipes,* another genera known to produce butyrate, in the rectum (R). Previous results indicate the important role of *Anerostipes* in maintaining gut homeostasis and controlling mucosal inflammation (Morrison and Preston, 2016). Moreover, we noted a strong relative enrichment of *Escherichia-Shigella* in patients with UC in the RC. In previous studies, invasive opportunistic pathogenic strains of *Escherichia* have been associated with worsened inflammation in DDS-induced colitis models in mice (Louis and Flint, 2017).

Co-analysing all samples from the lower GI tract revealed additional associations not captured by analyses of these sites individually (Figure 3). With this approach, and accounting for repeated sampling error, we identified absolute and/or relative depletion of numerous genera known to produce butyrate and/or propionate for UC and CD patients, consistent with results observed in stool samples (Shah et al., 2022). Production of these two short chain fatty acids has critical impacts upon the host’s physiology, including as a source of energy for intestinal epithelial cells, regulators of gene expression, signalling molecules, regulators of immune cell development and suppression of inflammation (Morrison and Preston, 2016; Louis and Flint, 2017).

PPI are often prescribed in patients with unexplained upper gastrointestinal symptoms, but their long-term effects on the small intestinal microbiome remains controversial (Shah et al., 2022) and is relatively understudied for the lower GI. In theory, by suppressing gastric acid secretion, the risk of gastrointestinal overgrowth and/or infections is increased. In that context, a recent systematic review and meta-analysis found an increased risk of bacterial overgrowth in the small intestine of PPI users (OR 1.71, 95% confidence interval 1.20–2.43) (Su et al., 2018). Imhann et al. found that the use of PPI was associated with a loss of alpha diversity and an increase in potential pathogenic bacterial genera *Enterococcus*, *Streptococcus*, *Staphylococcus* and *Escherichia* in faecal samples (Imhann et al., 2016). This contrasts with the results observed in the present study, where genera such as *Escherichia-Shigella* were noted to be depleted. This finding underscores the importance of profiling intestinal biopsies rather than relying solely on microbial analysis of fecal samples. In a previous study we also found that the bacterial load on the duodenal mucosa of PPI users was significantly higher compared to that in non-PPI users (Shah et al., 2022). Consistent with previous results, we observed a shift in the composition of the MAM in terms of beta diversity in accordance with PPI usage. Interestingly. *Faecalibacterium* abundance was increased in the lower GI tract of PPI users, noting that the genus was the most depleted in CD patients (Figure 4). In summation, PPI use appears to elicit impacts on the MAM of the lower GI despite the community profile in this region being greatly different to that of the upper GI (Mönnikes et al., 2011). Available evidence suggests that the effect on pH of PPIs is not significant in the lower GI (Gan et al., 1997). Despite this, changes in microbiome composition in the lower GI could be explained by the secretion of compounds to balance the pH changes elsewhere.

Even after achieving endoscopic remission, some IBD patients continue to experience debilitating GI symptoms. We examined the correlation between the gastrointestinal symptoms and the abundance of microorganisms of the MAM in patients with IBD. UC and CD patients with high gastrointestinal symptom scores, as assessed by SAGIS questionnaire, were noted to have a significantly differentiated MAM composition (beta diversity) in the upper and lower GI tract, in both relative and absolute terms. The strongest effect was the absolute and relative increase of *Faecalibacterium* abundance in the lower GI tract of patients with high gastrointestinal symptom scores. This result was unexpected given that *Faecalibacterium* was depleted in the MAM of IBD patients. This may be due to species/strain differences within this bacterial genus, and others, not unlike that for *Escherichia coli*, with some strains being commensal (i.e., *E. coli* [Nissle 1917]) (Adam et al., 2006) while others are pathogenic (i.e., *E. coli* [AIEC]) and have been associated with IBD (Palmela et al., 2018). However, our findings also raise the spectre that gut symptoms can persist beyond the positive impact(s) from medications on the microbiota and endoscopic remission. Given that the resolution of 16S amplicon analysis is limited to genus level, sophisticated techniques to investigate intestinal biopsies at the species or strain level are required to disentangle specific changes within these important lineages. However, routine use of shotgun metagenomic approaches will require technical advancements to counteract the problem of human signal overwhelming that of the microorganisms when DNA is extracted from mucosal biopsies.

It is well established that patients with DGBI exhibit significantly increased symptom responses to a standardised nutrient challenge (Shah et al., 2022). The severity of gastrointestinal symptoms after a nutrient challenge (Zhong et al., 2017) and responses to therapy are inherently linked to intestinal dysbiosis (Shah et al., 2022). We thus also assessed the link between the MAM and symptom response to a standardised nutrient challenge test as a measure of visceral sensory function. As compared control subjects, patients with IBD had numerically higher nutrient challenge scores (albeit not statistically significant—likely due to sample size constraints). However, we observed significant differentiation of the MAM composition (beta diversity) in those who had a higher symptom response score to the nutrient challenge test, in terms of both relative and absolute abundance (Figure 6). Lower levels of *Faecalibacterium* and other notable genera was associated with an increase in symptoms following the nutrient challenge (Figure 6B), whereas two genera which can play crucial roles in digestion, *Bacterioides* and *Bifidobacterium*, were enriched in individuals with elevated symptom response scores. The epigastric domain of the SAGIS was also examined to explore the correlation between self-reported “meal-related symptoms” and the MAM composition (Supplementary Figure S1), reinforcing that microbial composition may have a potential involvement in the development of a “sensitive gut” observed in patients with DGBI.

Our datasets have been produced using primers targeting the V6-V8 hypervariable region, consistent with our published studies of the mucosa-associated microbiota in humans (Shanahan et al., 2018, 2023; Zhong et al., 2017; Shah et al., 2022). The choice of primers for examining the duodenal microbiome was made around the time Illumina-based sequencing technology enabled the production of larger amplicons. This choice was also influenced by our early studies evaluating different primers for 16S rRNA profiling (Yu and Morrison, 2004). Re-evaluation of these primers with the “TestPrime” tool on the SILVA database website confirmed that they provide an excellent representation of taxonomic abundance across all Domains while minimizing host-derived amplicons, which is a key constraint in characterising the mucosa-associated microbiome Therefore, our selection of the V6-V8 primers in this study reflects both the recognition of the taxonomic range of microbial diversity across different gut segments and the desire to maintain consistency in taxonomy-based comparisons. We note that primers amplifying the V6-V8 region were recently evaluated by Abellan-Schneyder et al. (2021) using DNA from human microbiome samples and mock communities. The taxonomic assignments from the V6-V8 amplicons very comparable to the results produced using the V3-V4 primers, and the V6-V8 primers appeared more effective in recovering key mucosa-associated bacteria, such as *Verrucomicrobia* (*Akkermansia*). For these reasons, while the V3-V4 hypervariable region is widely (though not exclusively) targeted for 16S rRNA gene profiling in gut (stool) microbiomes, we are confident in the accuracy and interpretative context of our data from all gut segments.

In summary, our findings offer preliminary understanding of both the quantitative and spatial shifts in microbial taxa throughout the GI tract. The makeup of the MAM, especially in the lower GI tract, was revealed to influence various facets of health, treatment outcomes, and responses to symptoms. The consistent associations with specific bacterial genera underscore their significance as valuable targets for therapeutic interventions. Advancements in metagenomic techniques are poised to enhance our comprehension of the taxonomic and functional attributes of these therapeutic targets, enabling more precise and targeted therapeutic strategies.

## Data Availability

The datasets presented in this study can be found in online repositories. The names of the repository/repositories and accession number(s) can be found at: https://www.ncbi.nlm.nih.gov/, PRJNA1120972.

## Footnotes

^1^ https://github.com/biobakery/kneaddata
