# Supplementary material for: The biogeography of the mucosa-associated microbiome in health and disease
Source: Front Microbiol. 2024 Oct 14;15:1454910. doi: 10.3389/fmicb.2024.1454910 (PMC11513579; doi:10.3389/fmicb.2024.1454910)
Supplement: Supplementary file 2 [file Data_Sheet_1.docx]

**Supplementary file for the manuscript:** The biogeography of the mucosa-associated microbiome in health and disease.

**Legend of Figures:**

**Supplementary Figure 1.** Composition of the core microbiome at each site along the lower and upper gastrointestinal tracts of IBD patients. Heatmap values are expressed in log of the mean relative bacterial abundance from each site. Core microbiota present in 90%, 75% and 50% of participants are shown. Taxa lacking a g__ prefix were unable to be assigned at the genus level and represent family-level abundance. Samples were collected from the oesophagus (O), gastric antrum (G), duodenum (DU), terminal ileum (TI), right colon (RC) and rectum (R).

**Supplementary Figure 2.** Correlation of microbiome composition in UC and CD patients and gastrointestinal symptoms, as measured by the SAGIS epigastric scores, across the upper and lower GI tracts, measured in terms of relative and absolute abundance (lower GI). “High” corresponds to SAGIS scores ≥ 12, whereas “low” corresponds to SAGIS scores < 12. **A.** Multidimensional visualisation and PERMANOVA testing of microbial composition (beta diversity). **B.** Significantly enriched and depleted bacterial genera in UC and CD patients with high SAGIS (>12). *** P <0.001, ** P <0.01, * P <0.05.

**Legend of Tables:**

**Supplementary Table 1.** Demographic and clinical characteristics of IBD patients.

**Supplementary Table 2.** Demographic and clinical characteristics of controls.

**Supplementary Table 3.** Differentially abundant bacterial taxa associated with UC and CD across various gastrointestinal segments.

**Supplementary Table 4.** Differentially abundant bacterial taxa associated with proton pump inhibitor (PPI) usage.

**Supplementary Table 5.** Differentially abundant bacterial taxa associated with higher structured assessment of gastrointestinal symptom (SAGIS) scores.

**Supplementary Table 6.** Differentially abundant bacterial taxa associated with standardized nutrient challenge (SNC)scores.

**Legend of Files:**

**Supplementary File 1.** Clinical metadata used for the statistical analysis.

**Supplementary Table 1.** Demographic and clinical characteristics of IBD patients.

| **Groups** | **CD (n=31)** | **UC (n=44)** | **p value** |
| --- | --- | --- | --- |
| Age (years) | 43 (±12.3) | 45 (±14.2) | 0.4698 |
| Gender (Female), n (%) | 20 (64.5) | 22 (50) | 0.2444 |
| BMI (kg/m2) | 27.8 (±7.0) | 28.8 (±5.3) | 0.4696 |
| Current smoker, n (%) | **13 (41.9)** | **8 (18.2)** | **0.0113** |
| Ex-smoker, n (%) | 8 (25.8) | 7 (15.9) | 0.1047 |
| ***Medication:*** |  |  |  |
| PPI, n (%) | 3 (9.7) | 7 (15.9) | 0.5094 |
| Anti-Platelet, n (%) | 2 (6.5) | 4 (9.1) | >0.9999 |
| Anti-coagulant, n (%) | 1 (3.2) | 0 (0) | 0.4133 |
| NSAIDs, n (%) | 3 (9.7) | 1 (2.3) | 0.3003 |
| Iron Replacement Therapy, n (%) | 7 (22.6) | 4 (9.1) | 0.1831 |
| Creon, n (%) | 1 (3.2) | 1 (2.3) | >0.9999 |
| Prednisolone, n (%) | 3 (9.7) | 3 (6.8) | 0.6865 |
| Any Immunosuppressant, n (%) | **26 (83.9)** | **20 (45.4)** | **0.0008** |
| Biologics, n (%) | **17 (54.8)** | **7 (15.9)** | **0.0008** |
| AZA/6-MP, n (%) | 15 (48.4) | 13 (29.5) | 0.1454 |
| MTX, n (%) | **5 (16.1)** | **0 (0)** | **0.0098** |
| 5 ASA (Amino salicylates), n (%) | **2 (64.5)** | **28 (63.6)** | **<0.0001** |
| Anti-depressant, n (%) | 4 (12.9) | 5 (11.4) | >0.9999 |
| ***Clinical parameters:*** |  |  |  |
| **Time since diagnosis of IBD, years** |  |  |  |
| ≤   5 years | **9 (29.0)** | **23 (52.3)** | **0.0313** |
| ≤ 10 years | 15 (48.4) | 26 (59.1) | 0.2355 |
| ≥ 11 years | 16 (51.6) | 15 (34.1) | 0.2355 |
| Unknown | 0 (0) | 3 (6.8) | 0.2627 |
| **No of prior abdominal surgeries** |  |  |  |
| 1, n (%) | **11 (35.5)** | **5 (11.4)** | **0.0062** |
| 2, n (%) | **5 (16.1)** | **0 (0)** | **0.0028** |
| 3, n (%) | 0 (0) | 0 (0) | >0.9999 |
| 4 or more, n (%) | 1 (3.2) | 1 (2.3) | 0.4822 |
| None, n (%) | **14 (45.2)** | **38 (86.4)** | **0.0003** |
| **Extent of colitis** |  |  |  |
| Pancolitis, n (%) | NA | 18 (40.9) | NA |
| Left sided colitis, n (%) | NA | 13 (29.5) | NA |
| proctosigmoiditis, n (%) | NA | 1 (2.3) | NA |
| Colectomy with IPAA, n (%) | NA | NA | NA |
| **Montreal classification** |  |  |  |
| L1/L2/L3a, n | 12/0/19a | NA | NA |
| B1/B2/B3, n | 12/9/10 | NA | NA |
| Peri-anal disease, n (%) | 7 | NA | NA |
| **CDAI** | 55.5 (±51.6) | NA | NA |
| ***Biochemical and Haematological tests:*** |  |  |  |
| Hb, g/L * | 134.1 (±13.0) | 138.7 (±12.7) | 0.141 |
| MCV, fL* | 88.4 (±7.9) | 90.8 (±8.9) | 0.8198 |
| Ferritin, ng/mL* | 68.4 (±48.7) | 71.4 (±64.6) | 0.9933 |
| Transferrin Saturation, μg/dL* | **18.0 (8.0)** | **26.1 (±15.8)** | **0.0173** |
| Vitamin B12, pg/mL* | **277.9 (±246.2)** | **288.0 (±103.2)** | **0.0249** |
| CRP, mg/L* | 4.4 (±3.6) | 5.0 (±5.5) | 0.5484 |
| Albumin, mg/dl* | 40.6 (±3.7) | 41.4 (±3.7) | 0.1818 |
| anti-tissue transglutaminase IgA antibody positive, n (%) | 3.1 (±3.3) | 2.6 (±1.7) | 0.9846 |
| Helicobacter pylori serology, n (%) | 0 (0) | 5 (11.4) |  |
| Faecal calprotectin ug/g* | 154.4 (±154.2) | 147.9 (±250.6) | 0.1559 |
| ***Co-existing co-morbidities:*** |  |  |  |
| IBD related arthropathy, n (%) | 4 (12.9) | 2 (4.5) | 0.2237 |
| FGID overlay, n (%) | 4 (12.9) | 5 (11.4) | >0.9999 |
| Hypothyroidism, n (%) | 2 (6.5) | 2 (4.5) | >0.9999 |
| Pancreatic insufficiency, n (%) | 1 (3.2) | 0 (0) | 0.4133 |
| Eosinophilic Esophagitis, n (%) | 0 (0) | 1 (2.3) | >0.9999 |
| Depression, n (%) | 3 (9.7) | 5 (11.4) | >0.9999 |
| Insulin dependent diabetes mellitus, n (%) | 0 (0) | 1 (2.3) | >0.9999 |
| Type 2 diabetes mellitus, n (%) | 0 (0) | 3 (6.8) | 0.2627 |
| Ankylosing spondylitis, n (%) | 1 (3.2) | 1 (2.3) | >0.9999 |
| Rheumatoid arthritis, n (%) | 1 (3.2) | 0 (0) | 0.4133 |

*p* values were calculated using Fisher’s exact test (proportions), Mann-Whitney and Kruskal-Wallis or Student t-test when appropriate for comparisons of mean values. Statistically significant *p* values (*P<*0.05) are highlighted in **bold**. * Indicates values expressed as Mean (±Standard deviation). BMI; body mass index, PPI; Proton pump inhibitor, AZA; Azathioprine, 6-MP; 6-Mercaptopurine, MTX; Methotrexate, Hb; haemoglobin, MCV; Mean corpuscular volume, CRP; C-reactive protein, n; number, NSAIDs; Non-Steroidal Anti-inflammatory drugs, IRT; Iron Replacement Therapy, FGID; Functional gastrointestinal disorders, IBD; Inflammatory bowel disease, CD; Crohn’s disease, UC; ulcerative colitis, CDAI; Crohn’s disease activity index, IPAA; ileal pouch anal anastomosis, N/A; Not applicable, a; one patient also had upper gastrointestinal in addition to ileo-colonic CD.

**Supplementary Table 2.** Demographic and clinical characteristics of controls.

| **Groups** | **Iron deficiency with or without anaemia (n=27)** | **FOBT positive (n=32)** | **p value** |
| --- | --- | --- | --- |
| Age (years) * | 55 (±16.1) | 59 (±10.4) | 0.686 |
| Gender (Female), n (%) | 15 (55.6) | 13 (40.6) | 0.3017 |
| BMI (kg/m2) * | 29.3 (±6.6) | 26.6 (±5.4) | 0.1162 |
| Current smoker, n (%) | 3 (11.1) | 10 (31.3) | 0.1131 |
| Ex-smoker, n (%) | 7 (25.9) | 7 (21.9) | 0.7659 |
| ***Medication:*** |  |  |  |
| PPI, n (%) | 7 (25.9) | 2 (6.3) | 0.0659 |
| Anti-Platelets, n (%) | 3 (11.1) | 2 (6.3) | 0.6523 |
| Anti-coagulants, n (%) | 0 (0) | 0 (0) | >0.9999 |
| NSAIDs, n (%) | 1 (3.7) | 0 (0) | 0.4576 |
| Oral Iron Replacement Therapy, n (%) | **11 (40.7)** | **1 (3.1)** | **0.0006** |
| Any Immunosuppressant, n (%) | 0 (0) | 0 (0) | >0.9999 |
| Biologics, n (%) | 0 (0) | 0 (0) | >0.9999 |
| 5 ASA (Amino salicylates), n (%) | NA | 1 (3.1) | NA |
| Benzodiazepines, n (%) | NA | 5 (15.6) | NA |
| Anti-depressants, n (%) | NA | 0 (0) | NA |
| Thyroxine, n (%) | NA | 2 (6.3) | NA |
| Statins, n (%) | NA | 6 (18.8) | NA |
| Oral Hypoglycemic agents, n (%) | NA | 3 (9.4) | NA |
| ***Biochemical and Haematological tests:*** |  |  |  |
| Hb, g/L * | **133.4 (±11.4)** | **142.2 (±15.7)** | **0.034** |
| MCV, fL* | 86.3 (±7.4) | 96.0 (±30.3) | 0.1468 |
| Ferritin, ng/mL* | **92.2 (±147.8)** | **174.5 (±189.4)** | **0.0061** |
| Transferrin Saturation, μg/dL* | 20.5 (±13.9) | 37.1 (±52.5) | 0.0591 |
| Vitamin B12, pg/mL* | 312.6 (±298.1) | 288.2 (±147.5) | 0.706 |
| CRP, mg/L* | NA | 5.1 (±8.9) | NA |
| Albumin, mg/dl* | NA | 41.6 (±3.0) | NA |
| anti-tissue transglutaminase IgA antibody, positive n (%) | NA | 1.5 (±0.7) | NA |

*p* values were calculated using Fisher’s exact test (proportions), Mann-Whitney and Kruskal-Wallis or Student t-test when appropriate for comparisons of mean values. Statistically significant *p* values (*P<*0.05) are highlighted in **bold**. * Indicates values expressed as Mean (±Standard deviation). BMI; body mass index, PPI; Proton pump inhibitor, AZA; Azathioprine, 6-MP; 6-Mercaptopurine, MTX; Methotrexate, NSAIDs; Non-Steroidal Anti-inflammatory drugs, IRT; Iron Replacement Therapy, IV; Intravenous, Hb; haemoglobin, MCV; Mean corpuscular volume, CRP; C-reactive protein, n; number, FOBT; faecal occult blood test.

**Supplementary Table 3.** Differentially abundant bacterial taxa associated with UC and CD across various gastrointestinal segments.

| **Taxa** | **Metadata** | **Value** | **Coef** | **Pval** |
| --- | --- | --- | --- | --- |
| d__Bacteria;p__Bacteroidota;c__Bacteroidia;o__Bacteroidales;f__Barnesiellaceae;g__Coprobacter | R_absolute_abund | CD | 0.114654183 | 8.86E-11 |
| d__Bacteria;p__Desulfobacterota;c__Desulfovibrionia;o__Desulfovibrionales;f__Desulfovibrionaceae;g__Bilophila | R_relative_abund | CD | 0.089522162 | 4.67E-10 |
| d__Bacteria;p__Desulfobacterota;c__Desulfovibrionia;o__Desulfovibrionales;f__Desulfovibrionaceae;g__Bilophila | R_absolute_abund | CD | 0.05414475 | 1.83E-08 |
| d__Bacteria;p__Actinobacteriota;c__Coriobacteriia;o__Coriobacteriales;f__Eggerthellaceae;g__Eggerthella | R_absolute_abund | CD | 0.123140286 | 1.19E-07 |
| d__Bacteria;p__Firmicutes;c__Clostridia;o__Oscillospirales;f__Butyricicoccaceae;g__Butyricicoccus | R_absolute_abund | CD | 0.191692576 | 1.86E-07 |
| d__Bacteria;p__Firmicutes;c__Clostridia;o__Lachnospirales;f__Lachnospiraceae;g__;Ruminococcus;_torques_group | R_absolute_abund | CD | 0.158846173 | 2.10E-06 |
| d__Bacteria;p__Firmicutes;c__Clostridia;o__Lachnospirales;f__Lachnospiraceae;g__uncultured | R_absolute_abund | CD | 0.093888161 | 1.01E-05 |
| d__Bacteria;p__Firmicutes;c__Clostridia;o__Lachnospirales;f__Lachnospiraceae;g__Moryella | R_absolute_abund | CD | 0.062735197 | 1.15E-05 |
| d__Bacteria;p__Firmicutes;c__Clostridia;o__Lachnospirales;f__Lachnospiraceae;g__Dorea | R_absolute_abund | CD | 0.06779825 | 4.54E-05 |
| d__Bacteria;p__Firmicutes;c__Clostridia;o__Oscillospirales;f__Ruminococcaceae;g__Incertae_Sedis | R_absolute_abund | CD | 0.092877578 | 9.09E-05 |
| d__Bacteria;p__Firmicutes;c__Clostridia;o__Peptostreptococcales;Tissierellales;f__Peptostreptococcales;Tissierellales;g__Anaerococcus | RC_relative_abund | CD | 0.071644894 | 0.000115315 |
| d__Bacteria;p__Firmicutes;c__Bacilli;o__Erysipelotrichales;f__Erysipelatoclostridiaceae;g__Erysipelatoclostridium | R_absolute_abund | CD | 0.041877922 | 0.000198684 |
| d__Bacteria;p__Firmicutes;c__Clostridia;o__Lachnospirales;f__Lachnospiraceae;g__Lachnoclostridium | R_absolute_abund | CD | 0.064625273 | 0.000890412 |
| d__Bacteria;p__Bacteroidota;c__Bacteroidia;o__Bacteroidales;f__Bacteroidaceae;g__Bacteroides | R_absolute_abund | CD | 0.259631145 | 0.001244517 |
| d__Bacteria;p__Actinobacteriota;c__Coriobacteriia;o__Coriobacteriales;f__Eggerthellaceae;g__Eggerthella | TI_relative_abund | CD | 0.101326501 | 0.001253894 |
| d__Bacteria;p__Firmicutes;c__Clostridia;o__Peptostreptococcales;Tissierellales;f__Peptostreptococcales;Tissierellales;g__Anaerococcus | RC_absolute_abund | CD | 0.003455814 | 0.001255679 |
| d__Bacteria;p__Firmicutes;c__Clostridia;o__Lachnospirales;f__Lachnospiraceae;__ | R_absolute_abund | CD | 0.049073514 | 0.001266836 |
| d__Bacteria;p__Firmicutes;c__Clostridia;o__Peptostreptococcales;Tissierellales;f__Peptostreptococcales;Tissierellales;g__Anaerococcus | R_absolute_abund | CD | 0.023358557 | 0.001286537 |
| d__Bacteria;p__Bacteroidota;c__Bacteroidia;o__Bacteroidales;f__Barnesiellaceae;g__Coprobacter | RC_relative_abund | CD | 0.145060378 | 0.001471179 |
| d__Bacteria;p__Firmicutes;c__Clostridia;o__Oscillospirales;f__Oscillospiraceae;g__Flavonifractor | TI_absolute_abund | CD | 0.009740558 | 0.00151369 |
| d__Bacteria;p__Actinobacteriota;c__Coriobacteriia;o__Coriobacteriales;f__Eggerthellaceae;g__Eggerthella | TI_absolute_abund | CD | 0.01787095 | 0.001589044 |
| d__Bacteria;p__Firmicutes;c__Negativicutes;o__Veillonellales;Selenomonadales;f__Veillonellaceae;g__Megasphaera | G_relative_abund | CD | 0.055872115 | 0.002457477 |
| d__Bacteria;p__Proteobacteria;c__Gammaproteobacteria;o__Enterobacterales;f__Enterobacteriaceae;g__Escherichia;Shigella | R_absolute_abund | CD | 0.127753721 | 0.003157558 |
| d__Bacteria;p__Firmicutes;c__Negativicutes;o__Acidaminococcales;f__Acidaminococcaceae;g__Acidaminococcus | TI_relative_abund | CD | 0.047775513 | 0.003564989 |
| d__Bacteria;p__Firmicutes;c__Clostridia;o__Lachnospirales;f__Lachnospiraceae;g__Anaerostipes | RC_relative_abund | UC | -0.146074359 | 0.004074164 |
| d__Bacteria;p__Firmicutes;c__Clostridia;o__Oscillospirales;f__Oscillospiraceae;g__Flavonifractor | TI_relative_abund | CD | 0.056881688 | 0.004096251 |
| d__Bacteria;p__Bacteroidota;c__Bacteroidia;o__Bacteroidales;f__Rikenellaceae;g__Alistipes | R_absolute_abund | CD | 0.051860494 | 0.00472909 |
| d__Bacteria;p__Proteobacteria;c__Gammaproteobacteria;o__Burkholderiales;f__Sutterellaceae;g__Parasutterella | R_relative_abund | CD | 0.051719645 | 0.004812333 |
| d__Bacteria;p__Firmicutes;c__Clostridia;o__Lachnospirales;f__Lachnospiraceae;g__Moryella | R_relative_abund | CD | 0.068872932 | 0.004941198 |
| d__Bacteria;p__Firmicutes;c__Clostridia;o__Oscillospirales;f__Ruminococcaceae;g__UBA1819 | R_absolute_abund | CD | 0.029425601 | 0.005233117 |
| d__Bacteria;p__Proteobacteria;c__Gammaproteobacteria;o__Burkholderiales;f__Neisseriaceae;__ | G_relative_abund | CD | 0.01411 | 0.005502315 |
| d__Bacteria;p__Actinobacteriota;c__Coriobacteriia;o__Coriobacteriales;f__Eggerthellaceae;g__Eggerthella | R_relative_abund | CD | 0.149768021 | 0.005960796 |
| d__Bacteria;p__Bacteroidota;c__Bacteroidia;o__Bacteroidales;f__Barnesiellaceae;g__Coprobacter | R_relative_abund | CD | 0.105568839 | 0.00638551 |
| d__Bacteria;p__Desulfobacterota;c__Desulfovibrionia;o__Desulfovibrionales;f__Desulfovibrionaceae;g__Bilophila | RC_relative_abund | CD | 0.083412116 | 0.007493635 |
| d__Bacteria;p__Firmicutes;c__Negativicutes;o__Veillonellales;Selenomonadales;f__Veillonellaceae;g__Veillonella | RC_relative_abund | UC | 0.036776788 | 0.008088563 |
| d__Bacteria;p__Firmicutes;c__Clostridia;o__Lachnospirales;f__Lachnospiraceae;g__Moryella | RC_relative_abund | CD | 0.060753546 | 0.008441562 |
| d__Bacteria;p__Firmicutes;c__Clostridia;o__Oscillospirales;f__Ruminococcaceae;g__Faecalibacterium | RC_relative_abund | CD | -0.569610496 | 0.008668574 |
| d__Bacteria;p__Firmicutes;c__Clostridia;o__Lachnospirales;f__Lachnospiraceae;g__Tyzzerella | TI_relative_abund | CD | 0.063842933 | 0.010352869 |
| d__Bacteria;p__Firmicutes;c__Clostridia;o__Oscillospirales;f__Ruminococcaceae;g__Faecalibacterium | TI_relative_abund | CD | -0.312154962 | 0.011208947 |
| d__Bacteria;p__Firmicutes;c__Clostridia;o__Lachnospirales;f__Lachnospiraceae;g__Anaerostipes | TI_relative_abund | UC | -0.095148881 | 0.011425556 |
| d__Bacteria;p__Spirochaetota;c__Spirochaetia;o__Spirochaetales;f__Spirochaetaceae;g__Treponema | DU_relative_abund | CD | 0.02176206 | 0.0120747 |
| d__Bacteria;p__Firmicutes;c__Bacilli;o__Erysipelotrichales;f__Erysipelatoclostridiaceae;g__Erysipelatoclostridium | TI_absolute_abund | UC | 0.012261878 | 0.012087092 |
| d__Bacteria;p__Firmicutes;c__Clostridia;o__Lachnospirales;f__Lachnospiraceae;g__Lachnospira | RC_relative_abund | UC | 0.058818324 | 0.012402193 |
| d__Bacteria;p__Firmicutes;c__Clostridia;o__Lachnospirales;f__Lachnospiraceae;g__;Ruminococcus;_torques_group | RC_relative_abund | CD | 0.169950523 | 0.012430421 |
| d__Bacteria;p__Bacteroidota;c__Bacteroidia;o__Bacteroidales;f__Marinifilaceae;g__Butyricimonas | TI_relative_abund | UC | 0.019117553 | 0.015259996 |
| d__Bacteria;p__Proteobacteria;c__Gammaproteobacteria;o__Enterobacterales;f__Enterobacteriaceae;g__Escherichia;Shigella | RC_relative_abund | UC | 0.263837693 | 0.015769682 |
| d__Bacteria;p__Firmicutes;c__Clostridia;o__Lachnospirales;f__Lachnospiraceae;g__;Ruminococcus;_torques_group | R_relative_abund | CD | 0.138410812 | 0.016020935 |
| d__Bacteria;p__Firmicutes;c__Clostridia;o__Lachnospirales;f__Lachnospiraceae;g__Moryella | TI_absolute_abund | UC | 0.016383361 | 0.016533466 |
| d__Bacteria;p__Firmicutes;c__Bacilli;o__Erysipelotrichales;f__Erysipelatoclostridiaceae;g__Erysipelatoclostridium | RC_relative_abund | UC | 0.080859333 | 0.016582509 |
| d__Bacteria;p__Firmicutes;c__Clostridia;o__Lachnospirales;f__Lachnospiraceae;g__Anaerostipes | RC_relative_abund | CD | -0.209763639 | 0.017981443 |
| d__Bacteria;p__Firmicutes;c__Clostridia;o__Oscillospirales;f__Ruminococcaceae;g__Subdoligranulum | TI_relative_abund | CD | -0.236930706 | 0.018051062 |
| d__Bacteria;p__Firmicutes;c__Negativicutes;o__Acidaminococcales;f__Acidaminococcaceae;g__Phascolarctobacterium | RC_relative_abund | UC | -0.057264036 | 0.020518508 |
| d__Bacteria;p__Firmicutes;c__Clostridia;o__Oscillospirales;f__Ruminococcaceae;g__Faecalibacterium | R_relative_abund | CD | -0.396847056 | 0.020593269 |
| d__Bacteria;p__Firmicutes;c__Negativicutes;o__Acidaminococcales;f__Acidaminococcaceae;g__Phascolarctobacterium | R_relative_abund | UC | -0.043169777 | 0.021746242 |
| d__Bacteria;p__Proteobacteria;c__Gammaproteobacteria;o__Burkholderiales;f__Sutterellaceae;g__Sutterella | R_relative_abund | CD | -0.080218783 | 0.022258608 |
| d__Bacteria;p__Firmicutes;c__Clostridia;o__Lachnospirales;f__Lachnospiraceae;g__Anaerostipes | RC_absolute_abund | UC | -0.036658296 | 0.02318082 |
| d__Bacteria;p__Proteobacteria;c__Alphaproteobacteria;o__Sphingomonadales;f__Sphingomonadaceae;g__Sphingomonas | DU_relative_abund | CD | -0.035734013 | 0.024276385 |
| d__Bacteria;p__Firmicutes;c__Clostridia;o__Oscillospirales;f__Oscillospiraceae;g__Flavonifractor | TI_relative_abund | UC | 0.02049759 | 0.024662687 |
| d__Bacteria;p__Firmicutes;c__Clostridia;o__Clostridia_UCG;014;f__Clostridia_UCG;014;g__Clostridia_UCG;014 | DU_relative_abund | CD | -0.041277033 | 0.026605937 |
| d__Bacteria;p__Campilobacterota;c__Campylobacteria;o__Campylobacterales;f__Helicobacteraceae;g__Helicobacter | G_relative_abund | CD | -0.29179329 | 0.028218537 |
| d__Bacteria;p__Firmicutes;c__Clostridia;o__Lachnospirales;f__Lachnospiraceae;g__Moryella | RC_relative_abund | UC | 0.05382247 | 0.028530339 |
| d__Bacteria;p__Firmicutes;c__Clostridia;o__Oscillospirales;f__Ruminococcaceae;g__uncultured | TI_relative_abund | UC | 0.025231353 | 0.029084129 |
| d__Bacteria;p__Firmicutes;c__Clostridia;o__Christensenellales;f__Christensenellaceae;g__Christensenellaceae_R;7_group | R_absolute_abund | UC | -0.008400639 | 0.029270859 |
| d__Bacteria;p__Firmicutes;c__Clostridia;o__Oscillospirales;f__Oscillospiraceae;g__Flavonifractor | TI_absolute_abund | UC | 0.005473477 | 0.029490095 |
| d__Bacteria;p__Firmicutes;c__Bacilli;o__Erysipelotrichales;f__Erysipelotrichaceae;g__;Clostridium;_innocuum_group | TI_absolute_abund | CD | 0.027443176 | 0.030234633 |
| d__Bacteria;p__Firmicutes;c__Clostridia;o__Lachnospirales;f__Lachnospiraceae;g__Coprococcus | RC_absolute_abund | UC | -0.013058325 | 0.031661868 |
| d__Bacteria;p__Firmicutes;c__Clostridia;o__Lachnospirales;f__Lachnospiraceae;g__Moryella | TI_relative_abund | UC | 0.041107327 | 0.03188061 |
| d__Bacteria;p__Firmicutes;c__Clostridia;o__Lachnospirales;f__Lachnospiraceae;g__Anaerostipes | R_relative_abund | CD | -0.135113747 | 0.032398867 |
| d__Bacteria;p__Firmicutes;c__Negativicutes;o__Veillonellales;Selenomonadales;f__Veillonellaceae;g__Dialister | R_relative_abund | CD | 0.083707305 | 0.032588518 |
| d__Bacteria;p__Firmicutes;c__Clostridia;o__Oscillospirales;f__Ruminococcaceae;g__Ruminococcus | R_absolute_abund | UC | -0.015777008 | 0.032878198 |
| d__Bacteria;p__Firmicutes;c__Bacilli;o__Erysipelotrichales;f__Erysipelatoclostridiaceae;g__Erysipelatoclostridium | TI_relative_abund | UC | 0.039387634 | 0.034529191 |
| d__Bacteria;p__Firmicutes;c__Clostridia;o__Lachnospirales;f__Lachnospiraceae;g__Tyzzerella | TI_absolute_abund | CD | 0.007953716 | 0.03501093 |
| d__Bacteria;p__Fusobacteriota;c__Fusobacteriia;o__Fusobacteriales;f__Leptotrichiaceae;g__Leptotrichia | DU_relative_abund | CD | 0.051005915 | 0.03529149 |
| d__Bacteria;p__Bacteroidota;c__Bacteroidia;o__Bacteroidales;f__Prevotellaceae;g__Prevotella | R_relative_abund | UC | -0.096297432 | 0.035294175 |
| d__Bacteria;p__Firmicutes;c__Bacilli;o__Erysipelotrichales;f__Erysipelatoclostridiaceae;g__Erysipelatoclostridium | TI_relative_abund | CD | 0.04225973 | 0.035421271 |
| d__Bacteria;p__Firmicutes;c__Clostridia;o__Lachnospirales;f__Lachnospiraceae;g__uncultured | TI_relative_abund | CD | 0.067509798 | 0.03633548 |
| d__Bacteria;p__Firmicutes;c__Clostridia;o__Peptostreptococcales;Tissierellales;f__Peptostreptococcales;Tissierellales;g__Anaerococcus | R_relative_abund | UC | 0.032717022 | 0.036631539 |
| d__Bacteria;p__Firmicutes;c__Negativicutes;o__Acidaminococcales;f__Acidaminococcaceae;g__Acidaminococcus | R_absolute_abund | UC | 0.005141669 | 0.037396716 |
| d__Bacteria;p__Firmicutes;c__Clostridia;o__Oscillospirales;f__Ruminococcaceae;g__Ruminococcus | R_relative_abund | UC | -0.075541093 | 0.037427592 |
| d__Bacteria;p__Bacteroidota;c__Bacteroidia;o__Flavobacteriales;f__Weeksellaceae;g__Bergeyella | DU_relative_abund | CD | 0.011612258 | 0.037761378 |
| d__Bacteria;p__Bacteroidota;c__Bacteroidia;o__Bacteroidales;f__Rikenellaceae;g__Alistipes | TI_relative_abund | CD | 0.068973439 | 0.037995126 |
| d__Bacteria;p__Firmicutes;c__Clostridia;o__Oscillospirales;f__Oscillospiraceae;g__Colidextribacter | TI_relative_abund | CD | 0.095416542 | 0.038629798 |
| d__Bacteria;p__Firmicutes;c__Bacilli;o__Lactobacillales;__;__ | O_relative_abund | CD | -0.015267763 | 0.038702926 |
| d__Bacteria;p__Firmicutes;c__Clostridia;o__Oscillospirales;f__Ruminococcaceae;g__UBA1819 | TI_relative_abund | CD | 0.066628712 | 0.038822571 |
| d__Bacteria;p__Firmicutes;c__Clostridia;o__Oscillospirales;f__Ruminococcaceae;g__uncultured | R_absolute_abund | UC | -0.004928092 | 0.039178965 |
| d__Bacteria;p__Bacteroidota;c__Bacteroidia;o__Bacteroidales;f__Barnesiellaceae;g__Barnesiella | R_absolute_abund | UC | -0.011394529 | 0.040349102 |
| d__Bacteria;p__Firmicutes;c__Clostridia;o__Oscillospirales;f__Oscillospiraceae;g__uncultured | R_relative_abund | CD | 0.019617739 | 0.041839554 |
| d__Bacteria;p__Firmicutes;c__Clostridia;o__Lachnospirales;f__Lachnospiraceae;g__Blautia | TI_relative_abund | UC | -0.099403958 | 0.042188262 |
| d__Bacteria;p__Firmicutes;c__Negativicutes;o__Acidaminococcales;f__Acidaminococcaceae;g__Acidaminococcus | TI_absolute_abund | CD | 0.010213218 | 0.04219887 |
| d__Bacteria;p__Proteobacteria;c__Alphaproteobacteria;o__Rhizobiales;f__Xanthobacteraceae;g__Bradyrhizobium | DU_relative_abund | CD | 0.020433494 | 0.042567554 |
| d__Bacteria;p__Firmicutes;c__Clostridia;o__Clostridia_UCG;014;f__Clostridia_UCG;014;g__Clostridia_UCG;014 | TI_relative_abund | CD | -0.045894816 | 0.042601842 |
| d__Bacteria;p__Bacteroidota;c__Bacteroidia;o__Bacteroidales;f__Tannerellaceae;g__Tannerella | G_relative_abund | CD | 0.01825271 | 0.042825542 |
| d__Bacteria;p__Bacteroidota;c__Bacteroidia;o__Bacteroidales;f__Prevotellaceae;g__Prevotella | R_absolute_abund | UC | -0.014259057 | 0.044005292 |
| d__Bacteria;p__Firmicutes;c__Bacilli;o__Erysipelotrichales;f__Erysipelotrichaceae;g__;Clostridium;_innocuum_group | TI_relative_abund | CD | 0.076249344 | 0.044636784 |
| d__Bacteria;p__Bacteroidota;c__Bacteroidia;o__Flavobacteriales;f__Weeksellaceae;g__Cloacibacterium | O_relative_abund | CD | 0.021857721 | 0.045029758 |
| d__Bacteria;p__Cyanobacteria;c__Cyanobacteriia;o__Chloroplast;f__Chloroplast;g__Chloroplast | DU_relative_abund | CD | 0.035748359 | 0.045604714 |
| d__Bacteria;p__Firmicutes;c__Clostridia;o__Oscillospirales;f__Oscillospiraceae;g__Colidextribacter | TI_absolute_abund | CD | 0.010068202 | 0.046598804 |
| d__Bacteria;p__Firmicutes;c__Clostridia;o__Lachnospirales;f__Lachnospiraceae;g__Blautia | RC_absolute_abund | UC | -0.037110187 | 0.046622916 |
| d__Bacteria;p__Firmicutes;c__Clostridia;o__Lachnospirales;f__Lachnospiraceae;g__;Ruminococcus;_gnavus_group | R_absolute_abund | CD | 0.073622045 | 0.047422631 |
| d__Bacteria;p__Actinobacteriota;c__Coriobacteriia;o__Coriobacteriales;f__Coriobacteriaceae;g__Collinsella | TI_relative_abund | CD | -0.096683967 | 0.048307863 |
| d__Bacteria;p__Firmicutes;c__Clostridia;o__Lachnospirales;f__Lachnospiraceae;g__Coprococcus | R_relative_abund | CD | -0.059645002 | 0.04839724 |
| d__Bacteria;p__Firmicutes;c__Bacilli;o__Erysipelotrichales;f__Erysipelatoclostridiaceae;g__Erysipelatoclostridium | TI_absolute_abund | CD | 0.017648986 | 0.048773866 |

CD; Crohn’s disease, UC; ulcerative colitis, DU; duodenum, O; oesophagus, G; gastric, TI; terminal ileum, RC; right colon, R; rectum.

**Supplementary Table 4.** Differentially abundant bacterial taxa associated with proton pump inhibitor (PPI) usage.

| **Taxa** | **Metadata** | **Value** | **Coef** | **Pval** |
| --- | --- | --- | --- | --- |
| d__Bacteria;p__Actinobacteriota;c__Actinobacteria;o__Micrococcales;f__Cellulomonadaceae;g__Cellulomonas | PPI | upper_GI relative_abund | -0.133994207 | 2.62043E-07 |
| d__Bacteria;p__Firmicutes;c__Clostridia;o__Lachnospirales;f__Lachnospiraceae;g__Lachnospiraceae_NK4A136_group | PPI | lower_GI relative_abund | 0.027713618 | 3.84858E-05 |
| d__Bacteria;p__Actinobacteriota;c__Coriobacteriia;o__Coriobacteriales;f__Coriobacteriaceae;g__Collinsella | PPI | lower_GI relative_abund | 0.117799334 | 4.58076E-05 |
| d__Bacteria;p__Proteobacteria;c__Gammaproteobacteria;o__Pseudomonadales;f__Pseudomonadaceae;g__Pseudomonas | PPI | upper_GI relative_abund | -0.042212228 | 0.000395912 |
| d__Bacteria;p__Firmicutes;c__Clostridia;o__Monoglobales;f__Monoglobaceae;g__Monoglobus | PPI | lower_GI relative_abund | 0.033414269 | 0.000419729 |
| d__Bacteria;p__Proteobacteria;c__Gammaproteobacteria;o__Pasteurellales;f__Pasteurellaceae;g__Aggregatibacter | PPI | upper_GI relative_abund | 0.014034714 | 0.000558091 |
| d__Bacteria;p__Proteobacteria;c__Gammaproteobacteria;o__Pasteurellales;f__Pasteurellaceae;g__Actinobacillus | PPI | upper_GI relative_abund | 0.037638454 | 0.000897888 |
| d__Bacteria;p__Firmicutes;c__Clostridia;o__Monoglobales;f__Monoglobaceae;g__Monoglobus | PPI | lower_GI absolute_abund | 0.00708794 | 0.000907895 |
| d__Bacteria;p__Firmicutes;c__Bacilli;o__Erysipelotrichales;f__Erysipelatoclostridiaceae;g__Erysipelatoclostridium | PPI | lower_GI relative_abund | -0.037750964 | 0.003271639 |
| d__Bacteria;p__Patescibacteria;c__Saccharimonadia;o__Saccharimonadales;f__Saccharimonadaceae;g__Candidatus_Saccharimonas | PPI | upper_GI relative_abund | 0.008955287 | 0.00424572 |
| d__Bacteria;p__Proteobacteria;c__Alphaproteobacteria;o__Caulobacterales;f__Caulobacteraceae;g__uncultured | PPI | upper_GI relative_abund | -0.010198943 | 0.005237585 |
| d__Bacteria;p__Bacteroidota;c__Bacteroidia;o__Bacteroidales;f__Marinifilaceae;g__Odoribacter | PPI | lower_GI absolute_abund | 0.007035288 | 0.005430998 |
| d__Bacteria;p__Firmicutes;c__Clostridia;o__Oscillospirales;f__Ruminococcaceae;g__Faecalibacterium | PPI | lower_GI relative_abund | 0.183570971 | 0.005879188 |
| d__Bacteria;p__Firmicutes;c__Bacilli;o__Lactobacillales;f__Lactobacillaceae;g__Lactobacillus | PPI | upper_GI relative_abund | 0.022438003 | 0.011068165 |
| d__Bacteria;p__Firmicutes;c__Bacilli;o__Erysipelotrichales;f__Erysipelotrichaceae;g__Allobaculum | PPI | upper_GI relative_abund | 0.020586849 | 0.014683403 |
| d__Bacteria;p__Firmicutes;c__Clostridia;o__Lachnospirales;f__Lachnospiraceae;g__Fusicatenibacter | PPI | lower_GI relative_abund | -0.031690541 | 0.018571994 |
| d__Bacteria;p__Bacteroidota;c__Bacteroidia;o__Bacteroidales;f__Muribaculaceae;g__Muribaculaceae | PPI | upper_GI relative_abund | 0.03581035 | 0.018610609 |
| d__Bacteria;p__Actinobacteriota;c__Coriobacteriia;o__Coriobacteriales;f__Coriobacteriaceae;g__Collinsella | PPI | lower_GI absolute_abund | 0.025442228 | 0.021157619 |
| d__Bacteria;p__Firmicutes;c__Bacilli;o__Erysipelotrichales;f__Erysipelatoclostridiaceae;g__Erysipelatoclostridium | PPI | lower_GI absolute_abund | -0.008251039 | 0.021344116 |
| d__Bacteria;p__Proteobacteria;c__Gammaproteobacteria;o__Burkholderiales;f__Alcaligenaceae;__ | PPI | upper_GI relative_abund | 0.005420344 | 0.021501745 |
| d__Bacteria;p__Firmicutes;c__Clostridia;o__Oscillospirales;f__Ruminococcaceae;g__Negativibacillus | PPI | lower_GI relative_abund | 0.032760109 | 0.024846451 |
| d__Bacteria;p__Proteobacteria;c__Gammaproteobacteria;o__Burkholderiales;f__Neisseriaceae;g__Neisseria | PPI | upper_GI relative_abund | 0.029605348 | 0.025439306 |
| d__Bacteria;p__Firmicutes;c__Clostridia;o__Oscillospirales;f__;Eubacterium;_coprostanoligenes_group;g__;Eubacterium;_coprostanoligenes_group | PPI | lower_GI absolute_abund | 0.008148431 | 0.026141474 |
| d__Bacteria;p__Bacteroidota;c__Bacteroidia;o__Bacteroidales;f__Marinifilaceae;g__Odoribacter | PPI | lower_GI relative_abund | 0.023694027 | 0.0268147 |
| d__Bacteria;p__Bacteroidota;c__Bacteroidia;o__Bacteroidales;f__Barnesiellaceae;g__Coprobacter | PPI | lower_GI relative_abund | 0.022275604 | 0.026983735 |
| d__Bacteria;p__Proteobacteria;c__Gammaproteobacteria;o__Enterobacterales;f__Enterobacteriaceae;g__Escherichia;Shigella | PPI | lower_GI relative_abund | -0.113955184 | 0.032147673 |
| d__Bacteria;p__Patescibacteria;c__Saccharimonadia;o__Saccharimonadales;f__Saccharimonadales;g__Saccharimonadales | PPI | upper_GI relative_abund | 0.006533379 | 0.033845335 |
| d__Bacteria;p__Firmicutes;c__Bacilli;o__Lactobacillales;f__Streptococcaceae;g__Streptococcus | PPI | upper_GI relative_abund | 0.06023517 | 0.035483238 |
| d__Bacteria;p__Firmicutes;c__Clostridia;o__Lachnospirales;f__Lachnospiraceae;g__;Ruminococcus;_gnavus_group | PPI | lower_GI relative_abund | -0.06568165 | 0.03634554 |
| d__Bacteria;p__Firmicutes;c__Clostridia;o__Lachnospirales;f__Lachnospiraceae;g__Lachnospiraceae_NK4A136_group | PPI | lower_GI absolute_abund | 0.005066705 | 0.038447288 |
| d__Bacteria;p__Firmicutes;c__Clostridia;o__Oscillospirales;f__Ruminococcaceae;g__Faecalibacterium | PPI | lower_GI absolute_abund | 0.055421431 | 0.039417085 |
| d__Bacteria;p__Firmicutes;c__Clostridia;o__Oscillospirales;f__;Eubacterium;_coprostanoligenes_group;g__;Eubacterium;_coprostanoligenes_group | PPI | lower_GI relative_abund | 0.031629506 | 0.042518106 |
| d__Bacteria;p__Desulfobacterota;c__Desulfovibrionia;o__Desulfovibrionales;f__Desulfovibrionaceae;g__Bilophila | PPI | lower_GI relative_abund | -0.014513203 | 0.045530165 |

GI; gastrointestinal, DU; duodenum, O; oesophagus, G; gastric, TI; terminal ileum, RC; right colon, R; rectum.

**Supplementary Table 5.** Differentially abundant bacterial taxa associated with higher structured assessment of gastrointestinal symptom (SAGIS) scores.

| **Taxa** | **Metadata** | **Value** | **Coef** | **Pval** |
| --- | --- | --- | --- | --- |
| d__Bacteria;p__Bacteroidota;c__Bacteroidia;o__Bacteroidales;f__Prevotellaceae;g__Prevotella | lower_GI_relative_abund | High SAGIS | 0.064044738 | 6.44444E-05 |
| d__Bacteria;p__Bacteroidota;c__Bacteroidia;o__Bacteroidales;f__Prevotellaceae;g__Prevotella | upper_GI_relative_abund | High SAGIS | -0.148729979 | 0.000310578 |
| d__Bacteria;p__Proteobacteria;c__Gammaproteobacteria;o__Enterobacterales;f__Enterobacteriaceae;g__Escherichia;Shigella | lower_GI_relative_abund | High SAGIS | -0.153118256 | 0.00050501 |
| d__Bacteria;p__Bacteroidota;c__Bacteroidia;o__Bacteroidales;f__Prevotellaceae;g__Prevotella | lower_GI_absolute_abund | High SAGIS | 0.022215728 | 0.000549118 |
| d__Bacteria;p__Firmicutes;c__Clostridia;o__Peptostreptococcales;Tissierellales;f__Peptostreptococcaceae;g__Filifactor | upper_GI_relative_abund | High SAGIS | -0.023831512 | 0.00067154 |
| d__Bacteria;p__Bacteroidota;c__Bacteroidia;o__Bacteroidales;f__Barnesiellaceae;g__Coprobacter | lower_GI_relative_abund | High SAGIS | -0.029803797 | 0.001030576 |
| d__Bacteria;p__Firmicutes;c__Clostridia;o__Lachnospirales;f__Lachnospiraceae;g__Blautia | lower_GI_relative_abund | High SAGIS | 0.084766874 | 0.001081903 |
| d__Bacteria;p__Proteobacteria;c__Gammaproteobacteria;o__Enterobacterales;f__Enterobacteriaceae;g__Klebsiella | lower_GI_absolute_abund | High SAGIS | 0.036492233 | 0.00146132 |
| d__Bacteria;p__Bacteroidota;c__Bacteroidia;o__Bacteroidales;f__Prevotellaceae;g__Alloprevotella | upper_GI_relative_abund | High SAGIS | -0.098963099 | 0.001518545 |
| d__Bacteria;p__Proteobacteria;c__Gammaproteobacteria;o__Enterobacterales;f__Enterobacteriaceae;g__Escherichia;Shigella | lower_GI_absolute_abund | High SAGIS | -0.046364607 | 0.001846639 |
| d__Bacteria;p__Bacteroidota;c__Bacteroidia;o__Sphingobacteriales;f__Lentimicrobiaceae;g__Lentimicrobium | upper_GI_relative_abund | High SAGIS | -0.012434152 | 0.002380349 |
| d__Bacteria;p__Proteobacteria;c__Gammaproteobacteria;o__Enterobacterales;f__Enterobacteriaceae;g__Klebsiella | lower_GI_relative_abund | High SAGIS | 0.083580536 | 0.004205224 |
| d__Bacteria;p__Firmicutes;c__Clostridia;o__Oscillospirales;f__Ruminococcaceae;g__Ruminococcus | lower_GI_relative_abund | High SAGIS | 0.024510312 | 0.005136051 |
| d__Bacteria;p__Actinobacteriota;c__Actinobacteria;o__Bifidobacteriales;f__Bifidobacteriaceae;g__Bifidobacterium | lower_GI_absolute_abund | High SAGIS | 0.012951572 | 0.00594261 |
| d__Bacteria;p__Firmicutes;c__Clostridia;o__Peptostreptococcales;Tissierellales;f__Peptostreptococcaceae;g__Romboutsia | lower_GI_relative_abund | High SAGIS | 0.020649172 | 0.005978519 |
| d__Bacteria;p__Firmicutes;c__Clostridia;o__Lachnospirales;f__Lachnospiraceae;g__Fusicatenibacter | lower_GI_relative_abund | High SAGIS | 0.032168439 | 0.006421855 |
| d__Bacteria;p__Firmicutes;c__Clostridia;o__Lachnospirales;f__Lachnospiraceae;g__Blautia | lower_GI_absolute_abund | High SAGIS | 0.020435429 | 0.006728665 |
| d__Bacteria;p__Firmicutes;c__Negativicutes;o__Veillonellales;Selenomonadales;f__Veillonellaceae;g__Megasphaera | upper_GI_relative_abund | High SAGIS | -0.040282636 | 0.006770764 |
| d__Bacteria;p__Actinobacteriota;c__Coriobacteriia;o__Coriobacteriales;f__Atopobiaceae;g__Atopobium | upper_GI_relative_abund | High SAGIS | -0.056765428 | 0.009090899 |
| d__Bacteria;p__Proteobacteria;c__Gammaproteobacteria;o__Burkholderiales;f__Rhodocyclaceae;g__Dechloromonas | upper_GI_relative_abund | High SAGIS | 0.018707932 | 0.010450746 |
| d__Bacteria;p__Firmicutes;c__Clostridia;o__Oscillospirales;f__Ruminococcaceae;g__uncultured | lower_GI_absolute_abund | High SAGIS | -0.004116224 | 0.011018039 |
| d__Bacteria;p__Firmicutes;c__Clostridia;o__Oscillospirales;f__Ruminococcaceae;g__Faecalibacterium | lower_GI_relative_abund | High SAGIS | 0.154101362 | 0.013654234 |
| d__Bacteria;p__Firmicutes;c__Negativicutes;o__Veillonellales;Selenomonadales;f__Selenomonadaceae;g__Megamonas | lower_GI_relative_abund | High SAGIS | -0.016009023 | 0.01439645 |
| d__Bacteria;p__Firmicutes;c__Clostridia;o__Oscillospirales;f__Ruminococcaceae;g__Ruminococcus | lower_GI_absolute_abund | High SAGIS | 0.004603852 | 0.018570891 |
| d__Bacteria;p__Firmicutes;c__Clostridia;o__Christensenellales;f__Christensenellaceae;g__Christensenellaceae_R;7_group | lower_GI_relative_abund | High SAGIS | 0.016597833 | 0.02069469 |
| d__Bacteria;p__Firmicutes;c__Clostridia;o__Lachnospirales;f__Lachnospiraceae;g__Fusicatenibacter | lower_GI_absolute_abund | High SAGIS | 0.006295422 | 0.02259412 |
| d__Bacteria;p__Firmicutes;c__Clostridia;o__Lachnospirales;f__Lachnospiraceae;g__Tyzzerella | lower_GI_absolute_abund | High SAGIS | -0.010029589 | 0.02420504 |
| d__Bacteria;p__Firmicutes;c__Clostridia;o__Lachnospirales;f__Lachnospiraceae;g__Coprococcus | lower_GI_relative_abund | High SAGIS | 0.025059168 | 0.024213214 |
| d__Bacteria;p__Firmicutes;c__Clostridia;o__Christensenellales;f__Christensenellaceae;g__Christensenellaceae_R;7_group | lower_GI_absolute_abund | High SAGIS | 0.005149215 | 0.024984277 |
| d__Bacteria;p__Proteobacteria;c__Gammaproteobacteria;o__Burkholderiales;f__Neisseriaceae;g__Neisseria | upper_GI_relative_abund | High SAGIS | 0.058331942 | 0.025464443 |
| d__Bacteria;p__Firmicutes;c__Clostridia;o__Monoglobales;f__Monoglobaceae;g__Monoglobus | lower_GI_absolute_abund | High SAGIS | 0.004662755 | 0.027339658 |
| d__Bacteria;p__Bacteroidota;c__Bacteroidia;o__Bacteroidales;f__Barnesiellaceae;g__Coprobacter | lower_GI_absolute_abund | High SAGIS | -0.007387228 | 0.031989018 |
| d__Bacteria;p__Firmicutes;c__Clostridia;o__Oscillospirales;f__Ruminococcaceae;g__Faecalibacterium | lower_GI_absolute_abund | High SAGIS | 0.054164 | 0.032332941 |
| d__Bacteria;p__Firmicutes;c__Clostridia;o__Peptostreptococcales;Tissierellales;f__Peptostreptococcaceae;g__Intestinibacter | lower_GI_relative_abund | High SAGIS | -0.053968479 | 0.032735509 |
| d__Bacteria;p__Firmicutes;c__Negativicutes;o__Acidaminococcales;f__Acidaminococcaceae;g__Phascolarctobacterium | lower_GI_relative_abund | High SAGIS | 0.018647073 | 0.03453966 |
| d__Bacteria;p__Firmicutes;c__Negativicutes;o__Veillonellales;Selenomonadales;f__Veillonellaceae;g__Dialister | lower_GI_absolute_abund | High SAGIS | 0.008890603 | 0.035264825 |
| d__Bacteria;p__Firmicutes;c__Clostridia;o__Oscillospirales;f__;Eubacterium;_coprostanoligenes_group;g__;Eubacterium;_coprostanoligenes_group | lower_GI_relative_abund | High SAGIS | 0.022170082 | 0.039135252 |
| d__Bacteria;p__Firmicutes;c__Clostridia;o__Peptostreptococcales;Tissierellales;f__Peptostreptococcaceae;g__Romboutsia | lower_GI_absolute_abund | High SAGIS | 0.005199281 | 0.04013228 |
| d__Bacteria;p__Firmicutes;c__Clostridia;o__Monoglobales;f__Monoglobaceae;g__Monoglobus | lower_GI_relative_abund | High SAGIS | 0.01952259 | 0.040707624 |
| d__Bacteria;p__Actinobacteriota;c__Actinobacteria;o__Bifidobacteriales;f__Bifidobacteriaceae;g__Bifidobacterium | lower_GI_relative_abund | High SAGIS | 0.030080191 | 0.045118442 |
| d__Bacteria;p__Firmicutes;c__Negativicutes;o__Acidaminococcales;f__Acidaminococcaceae;g__Acidaminococcus | lower_GI_relative_abund | High SAGIS | -0.01560877 | 0.045660801 |
| d__Bacteria;p__Proteobacteria;c__Gammaproteobacteria;o__Enterobacterales;f__Enterobacteriaceae;__ | lower_GI_relative_abund | High SAGIS | -0.05254509 | 0.045814485 |
| d__Bacteria;p__Firmicutes;c__Clostridia;o__Lachnospirales;f__Lachnospiraceae;g__;Ruminococcus;_gauvreauii_group | lower_GI_relative_abund | High SAGIS | -0.008910678 | 0.045946501 |
| d__Bacteria;p__Proteobacteria;c__Gammaproteobacteria;o__Burkholderiales;f__Comamonadaceae;g__Pelomonas | upper_GI_relative_abund | High SAGIS | 0.014642137 | 0.046323232 |
| d__Bacteria;p__Firmicutes;c__Clostridia;o__Oscillospirales;f__Oscillospiraceae;g__UCG;002 | lower_GI_relative_abund | High SAGIS | 0.009162591 | 0.047254812 |
| d__Bacteria;p__Firmicutes;c__Clostridia;o__Peptostreptococcales;Tissierellales;f__Peptostreptococcaceae;__ | lower_GI_relative_abund | High SAGIS | 0.014399975 | 0.049776536 |
| d__Bacteria;p__Firmicutes;c__Clostridia;o__Oscillospirales;f__Oscillospiraceae;g__Flavonifractor | lower_GI_absolute_abund | High SAGIS | -0.003511694 | 0.049780333 |
| d__Bacteria;p__Firmicutes;c__Clostridia;o__Peptostreptococcales;Tissierellales;f__Anaerovoracaceae;g__;Eubacterium;_brachy_group | upper_GI_relative_abund | High SAGIS | -0.012011304 | 0.049801256 |

GI; gastrointestinal, DU; duodenum, O; oesophagus, G; gastric, TI; terminal ileum, RC; right colon, R; rectum.

**Supplementary Table 6.** Differentially abundant bacterial taxa associated with standardized nutrient challenge (SNC)scores.

| **Taxa** | **Metadata** | **Value** | **Coef** | **Pval** |
| --- | --- | --- | --- | --- |
| d__Bacteria;p__Actinobacteriota;c__Actinobacteria;o__Bifidobacteriales;f__Bifidobacteriaceae;g__Bifidobacterium | lower_GI_relative_abund | NC_total_score | 0.046212838 | 1.33E-07 |
| d__Bacteria;p__Firmicutes;c__Clostridia;o__Monoglobales;f__Monoglobaceae;g__Monoglobus | lower_GI_absolute_abund | NC_total_score | 0.00454024 | 0.000102253 |
| d__Bacteria;p__Firmicutes;c__Bacilli;o__Lactobacillales;f__Streptococcaceae;g__Streptococcus | lower_GI_absolute_abund | NC_total_score | 0.003949052 | 0.000182741 |
| d__Bacteria;p__Actinobacteriota;c__Actinobacteria;o__Bifidobacteriales;f__Bifidobacteriaceae;g__Bifidobacterium | lower_GI_absolute_abund | NC_total_score | 0.011124568 | 0.000238495 |
| d__Bacteria;p__Proteobacteria;c__Gammaproteobacteria;o__Enterobacterales;f__Enterobacteriaceae;__ | lower_GI_relative_abund | NC_total_score | -0.051764843 | 0.001524473 |
| d__Bacteria;p__Proteobacteria;c__Gammaproteobacteria;o__Pasteurellales;f__Pasteurellaceae;g__Haemophilus | lower_GI_relative_abund | NC_total_score | 0.010108985 | 0.001605604 |
| d__Bacteria;p__Firmicutes;c__Clostridia;o__Clostridia_UCG;014;f__Clostridia_UCG;014;g__Clostridia_UCG;014 | lower_GI_relative_abund | NC_total_score | 0.013854514 | 0.004704443 |
| d__Bacteria;p__Firmicutes;c__Clostridia;o__Oscillospirales;f__Ruminococcaceae;g__Faecalibacterium | lower_GI_relative_abund | NC_total_score | -0.064695303 | 0.00472507 |
| d__Bacteria;p__Firmicutes;c__Clostridia;o__Clostridia_UCG;014;f__Clostridia_UCG;014;g__Clostridia_UCG;014 | lower_GI_absolute_abund | NC_total_score | 0.004206457 | 0.005712319 |
| d__Bacteria;p__Proteobacteria;c__Gammaproteobacteria;o__Burkholderiales;f__Sutterellaceae;g__Sutterella | lower_GI_absolute_abund | NC_total_score | 0.01005848 | 0.007591115 |
| d__Bacteria;p__Firmicutes;c__Clostridia;o__Lachnospirales;f__Lachnospiraceae;g__Lachnospiraceae_NK4A136_group | lower_GI_absolute_abund | NC_total_score | 0.004076156 | 0.007677216 |
| d__Bacteria;p__Firmicutes;c__Clostridia;o__Lachnospirales;f__Lachnospiraceae;g__GCA;900066575 | lower_GI_relative_abund | NC_total_score | 0.012318809 | 0.009045533 |
| d__Bacteria;p__Firmicutes;c__Clostridia;o__Lachnospirales;f__Lachnospiraceae;g__Dorea | lower_GI_absolute_abund | NC_total_score | 0.005643991 | 0.009080109 |
| d__Bacteria;p__Firmicutes;c__Clostridia;o__Lachnospirales;f__Lachnospiraceae;g__GCA;900066575 | lower_GI_absolute_abund | NC_total_score | 0.003999346 | 0.009990012 |
| d__Bacteria;p__Proteobacteria;c__Gammaproteobacteria;o__Enterobacterales;f__Enterobacteriaceae;__ | lower_GI_absolute_abund | NC_total_score | -0.010031717 | 0.010311303 |
| d__Bacteria;p__Firmicutes;c__Negativicutes;o__Veillonellales;Selenomonadales;f__Veillonellaceae;g__Veillonella | lower_GI_absolute_abund | NC_total_score | 0.001993473 | 0.012192793 |
| d__Bacteria;p__Proteobacteria;c__Gammaproteobacteria;o__Pasteurellales;f__Pasteurellaceae;g__Haemophilus | lower_GI_absolute_abund | NC_total_score | 0.004165049 | 0.012241251 |
| d__Bacteria;p__Bacteroidota;c__Bacteroidia;o__Bacteroidales;f__Marinifilaceae;g__Odoribacter | lower_GI_absolute_abund | NC_total_score | 0.002899047 | 0.013532689 |
| d__Bacteria;p__Firmicutes;c__Clostridia;o__Lachnospirales;f__Lachnospiraceae;g__Anaerostipes | lower_GI_relative_abund | NC_total_score | -0.027108575 | 0.013922918 |
| d__Bacteria;p__Firmicutes;c__Clostridia;o__Oscillospirales;f__Oscillospiraceae;g__UCG;002 | lower_GI_absolute_abund | NC_total_score | 0.002124262 | 0.015711899 |
| d__Bacteria;p__Firmicutes;c__Clostridia;o__Oscillospirales;f__Ruminococcaceae;g__Subdoligranulum | lower_GI_absolute_abund | NC_total_score | 0.011136409 | 0.016617877 |
| d__Bacteria;p__Firmicutes;c__Clostridia;o__Christensenellales;f__Christensenellaceae;g__Christensenellaceae_R;7_group | lower_GI_absolute_abund | NC_total_score | 0.004817459 | 0.017149598 |
| d__Bacteria;p__Firmicutes;c__Clostridia;o__Lachnospirales;f__Lachnospiraceae;g__Roseburia | lower_GI_relative_abund | NC_total_score | 0.017369431 | 0.020601531 |
| d__Bacteria;p__Bacteroidota;c__Bacteroidia;o__Bacteroidales;f__Barnesiellaceae;g__Barnesiella | lower_GI_relative_abund | NC_total_score | -0.013613461 | 0.021269149 |
| d__Bacteria;p__Proteobacteria;c__Gammaproteobacteria;o__Burkholderiales;f__Sutterellaceae;g__Sutterella | lower_GI_relative_abund | NC_total_score | 0.018154013 | 0.021656078 |
| d__Bacteria;p__Firmicutes;c__Clostridia;o__Monoglobales;f__Monoglobaceae;g__Monoglobus | lower_GI_relative_abund | NC_total_score | 0.009909205 | 0.022009524 |
| d__Bacteria;p__Firmicutes;c__Clostridia;o__Lachnospirales;f__Lachnospiraceae;g__Lachnospiraceae_NK4A136_group | lower_GI_relative_abund | NC_total_score | 0.007396358 | 0.022854522 |
| d__Bacteria;p__Firmicutes;c__Negativicutes;o__Acidaminococcales;f__Acidaminococcaceae;g__Phascolarctobacterium | lower_GI_absolute_abund | NC_total_score | 0.004364892 | 0.024398816 |
| d__Bacteria;p__Firmicutes;c__Clostridia;o__Oscillospirales;f__Ruminococcaceae;g__Subdoligranulum | lower_GI_relative_abund | NC_total_score | 0.036947982 | 0.028503185 |
| d__Bacteria;p__Actinobacteriota;c__Coriobacteriia;o__Coriobacteriales;f__Eggerthellaceae;g__Senegalimassilia | lower_GI_relative_abund | NC_total_score | 0.010544003 | 0.032757401 |
| d__Bacteria;p__Firmicutes;c__Clostridia;o__Oscillospirales;f__Ruminococcaceae;g__Incertae_Sedis | lower_GI_absolute_abund | NC_total_score | 0.005924442 | 0.034211995 |
| d__Bacteria;p__Firmicutes;c__Clostridia;o__Oscillospirales;f__Ruminococcaceae;g__Incertae_Sedis | lower_GI_relative_abund | NC_total_score | 0.018534013 | 0.035329104 |
| d__Bacteria;p__Actinobacteriota;c__Coriobacteriia;o__Coriobacteriales;f__Eggerthellaceae;g__Senegalimassilia | lower_GI_absolute_abund | NC_total_score | 0.003968111 | 0.036877866 |
| d__Bacteria;p__Firmicutes;c__Clostridia;o__Lachnospirales;f__Lachnospiraceae;g__Lachnospira | lower_GI_absolute_abund | NC_total_score | 0.001360934 | 0.038588673 |
| d__Bacteria;p__Proteobacteria;c__Gammaproteobacteria;o__Enterobacterales;f__Enterobacteriaceae;g__Escherichia;Shigella | lower_GI_absolute_abund | NC_total_score | -0.007910808 | 0.040093882 |
| d__Bacteria;p__Bacteroidota;c__Bacteroidia;o__Bacteroidales;f__Bacteroidaceae;g__Bacteroides | lower_GI_relative_abund | NC_total_score | 0.040882473 | 0.043331995 |

GI; gastrointestinal, DU; duodenum, O; oesophagus, G; gastric, TI; terminal ileum, RC; right colon, R; rectum
