# Supplementary figures and images for: The biogeography of the mucosa-associated microbiome in health and disease
Source: Front Microbiol. 2024 Oct 14;15:1454910. doi: 10.3389/fmicb.2024.1454910 (PMC11513579; doi:10.3389/fmicb.2024.1454910)

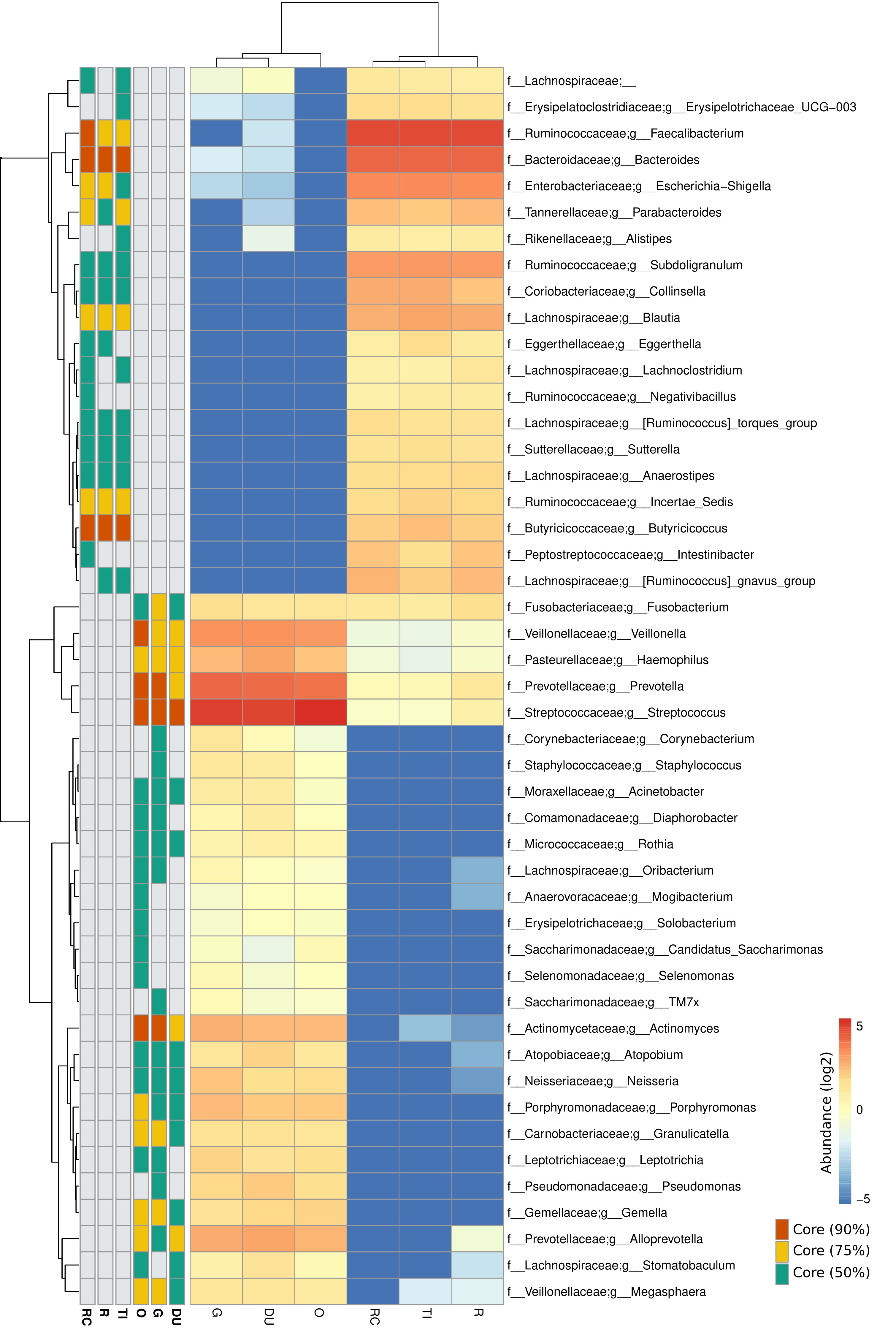

Supplement: Supplementary file 3 [file Image_1.JPEG]

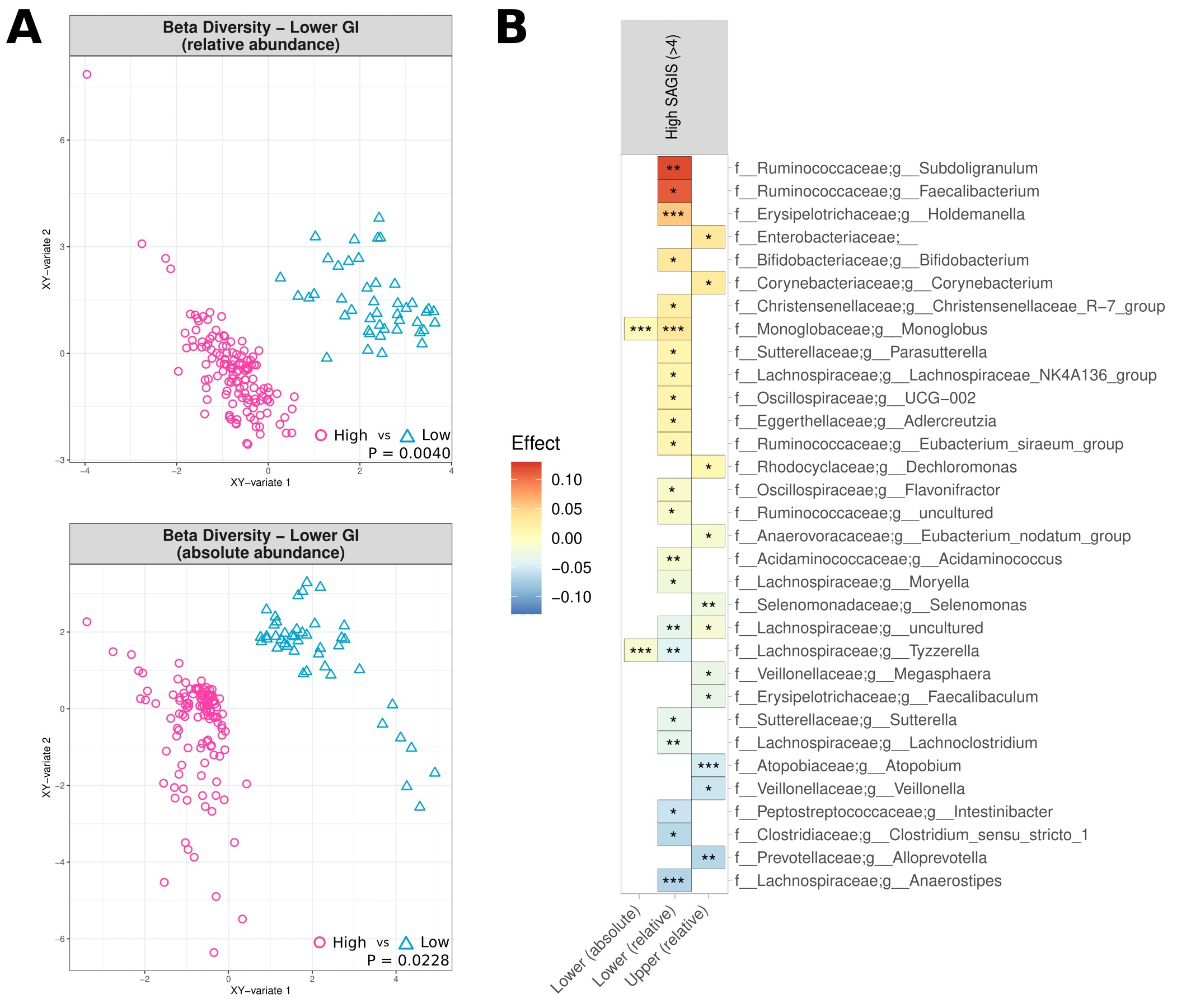

Supplement: Supplementary file 4 [file Image_2.JPEG]
